# Porosity Tuning in Soft-templated Mesoporous Silica: The Influence of Block Copolymer Composition and Concentration

**DOI:** 10.1021/acs.langmuir.5c02750

**Published:** 2025-11-13

**Authors:** Lysander Q. Wagner, Frederik Breckwoldt, Xiaohui Huang, Christian Kübel, Xiaoyin Cheng, Katja Schladitz, Bernd M. Smarsly

**Affiliations:** † Institute of Physical Chemistry, Justus Liebig University, Heinrich-Buff-Ring 17, Giessen D-35392, Germany; ‡ Center of Materials Research, Justus Liebig University, Heinrich-Buff-Ring 16, Giessen D-35392, Germany; § Institute of Nanotechnology, Karlsruhe Institute of Technology, Hermann-von-Helmholtz-Platz 1, Eggenstein-Leopoldshafen D-76344, Germany; ∥ Department of Materials and Earth Science, Technical University Darmstadt, Peter-Grünberg-Straße 2, Darmstadt D-64287, Germany; ⊥ Karlsruhe Nano Micro Facility, Karlsruhe Institute of Technology, Hermann-von-Helmholtz-Platz 1, Eggenstein-Leopoldshafen D-76344, Germany; # Fraunhofer Institute for Industrial Mathematics, Fraunhofer-Platz 1, Kaiserslautern D-67663, Germany

## Abstract

Macroscopic properties of mesoporous metal oxides depend on the mesopore architecture, i.e., the pore size, wall thickness, and pore connectivity. Consequently, rational preparation protocols and deep knowledge of the templating mechanism are required for systematic porosity–property studies and the intentional synthesis of an optimized pore morphology. In this work, we thus prepared a library of 17 poly­(ethylene oxide)-*block*-poly­(hexyl acrylate) (PEO-*b*-PHA) block copolymers of varying PEO and PHA block lengths to quantitatively deduce the effect of the individual block lengths on the mesopore size of the templated silica. The block length of the hydrophobic PHA block in the micelle core showed to enable a pore size tuning between 10 and 80 nm according to electron microscopy, physisorption, and small-angle X-ray scattering. In contrast, varying the PEO block length did not alter the pore size, but revealed that a sufficiently large PEO block is required to ensure ordered spherical mesopores. Electron tomography confirmed a spherical pore geometry and a systematic decrease in pore wall thickness upon increasing the template concentration (i.e., template-to-silica ratio) during soft templating. A statistical in-depth analysis by tomography demonstrated that this wall size decrease is accompanied by an improved pore connectivity (e.g., in terms of the coordination number of adjacent pores) and an increasing pore size. The pore size increase originates from a partial PEO collapse on the micelle core based on a pore volume analysis and occurs only above a certain threshold concentration of block copolymer. We demonstrated that this concentration can be elevated by applying soft templates featuring shorter PEO blocks, which extend the regime of wall size tuning under preservation of pore dimension and shape. Overall, these insights provide a guideline on how to tailor the pore size, wall thickness, and pore connectivity of mesoporous metal oxides and enable systematic studies concerning the optimum porosity, e.g., for electrocatalytic applications to maximize stability and activity.

## Graphical abstract

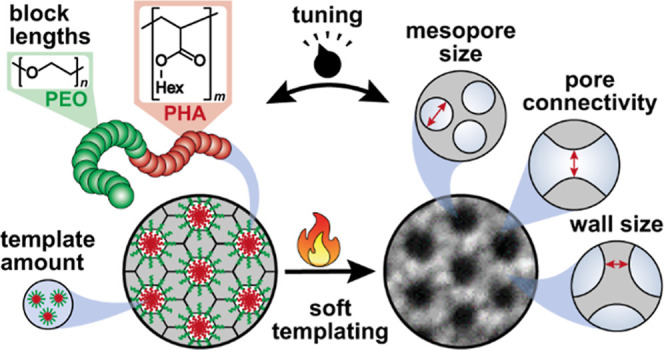

## Introduction

Ordered mesoporous powders and thin films have attracted great attention in the past 30 years. As model materials with a uniform structure, they helped to understand and advance characterization methods such as gas physisorption,^1–7^ mercury intrusion porosimetry,^8^ and ellipsometric porosimetry,^9,10^ which now enable a reliable and standardized analysis of materials with a disordered pore space. Thus, a systematic synthesis of well-ordered structures is required to expand routine techniques for pore analysis. In application, mesoporous materials take advantage of their high surface (or interface) area and large pore volume, boosting the performance of catalysts,^11–15^ adsorbents,^16^ gas sensors,^17,18^ coatings,^19^ and energy materials.^20–22^ Hereby, it is important to mention that macroscopic properties like conductivity, catalytic activity, capacitance, and both thermal and mass transport show a dependency on mesoscopic parameters, namely, pore size,^23–29^ wall thickness,^30^ and pore connectivity.^22,31–33^ Consequently, dedicated preparation protocols and closer insights into the templating behavior are required to (1) investigate porosity–property relationships systematically and (2) generate an optimized pore morphology for a desired property precisely and deliberately.

Tuning of the mesopore size is often achieved by adjusting synthesis temperature,^34–36^ varying the amount^34,37^ and kind^38^ of acid used as a sol–gel catalyst, and using additives^12,39^ or swelling agents^35,40–42^ within the soft templating process. Such modifications in the synthesis protocol, however, might result in the alteration of the final skeleton material as well, e.g., its crystallite size.^43^ To keep the skeleton material ideally untouched and to vary only the pore morphology, tuning the soft template appears to be a viable option. Especially, if block copolymers are applied as structure-directing agents, variation of the block lengths represents, at first glance, a straightforward approach to adjust the pore size without changing the chemical nature of the components in the synthesis. Concerning the pore size regime, a range of 30–70 nm is of particular interest, still providing a high surface area but avoiding possible diffusion limitations of small pore networks.^44,45^ Here, diblock copolymers of poly­(ethylene oxide) and poly­(hexyl acrylate) (PEO-*b*-PHA) appear especially suitable and superior due to their facile synthesis^46,47^ and the ability to introduce large mesopores of around 50 nm in diameter.^22,46,48^ Although PEO-*b*-PHA has already been used for soft templating, a precise relationship between PEO and PHA block lengths and the resulting mesopore size would further spur this field. Similarly, the question arises, which pore size region can be covered with this polymer class at all.

Besides the pore size, the pore wall thickness features a further major porosity parameter, e.g., by restricting crystallite growth within the pore wall. Tailoring the pore wall thickness is accomplished in most cases by changing the ratio between precursor and soft template.^12,47–50^ This modification is only possible if soft templating follows an evaporation-induced self-assembly (EISA)^51–53^ mechanism: As shown in Figure 1, the amphiphilic soft template forms micelles, which arrange themselves in a closely packed pattern during the proceeding solvent evaporation. In the space between the micelles, the (metal oxide) precursor condenses to a gel. A calcination step decomposes the template and converts the micelle pattern to an array of mesopores. If the soft template micelles are evenly dispersed within the matrix of the condensed precursor, the precursor-to-template ratio governs the micelle-to-micelle and thus the pore-to-pore distance, i.e., the pore wall thickness (as long as no phase separation occurs, i.e., precipitation of the template). Contrarily, following an evaporation-induced aggregating assembly (EIAA)^54^ mechanism, micelles and precursors assemble in dispersion (Figure 1) and hence do not allow a wall size tuning by varying the template amount. While Stefik and co-workers reported an independent adjustment of pore and wall size for soft templating of Nb_2_O_5_ with PEO-*b*-PHA,^47,48,50^ we observed an increase in pore size in silica following an EISA approach upon increasing the template amount.^46^ Shao et al. observed a similar trend with Pluronic polymer P123 used for soft templating of titanium dioxide.^25^ Also, studies on poly­(isoprene)-*block*-poly­(ethylene oxide) confirm an influence of the metal precursor amount on the local PEO environment, yielding different mesopore sizes and morphologies.^55–57^ This apparent contradiction demonstrates that the influence of the template-to-precursor ratio is complex and evokes the question of how the soft template amount affects the pore space, i.e., pore size, wall size, and pore connectivity, over a broad range for a given block copolymer (BCP). Indeed, micelle kinetics and the solvent used to disperse them play an important role, as equilibration of micelles by polymer chain exchange depends on the block length^58^ *N* and the interaction parameter^59^ χ between the solvent and the solvophobic block in the micelle core. Thus, depending on the χ *N* barrier for chain exchange (being very low in case of Pluronic P123 and very high for PEO-*b*-PHA, for instance, when presuming a hydrophilic solvent), BCP micelles can equilibrate according to their environment (dynamic micelles) or are kinetically trapped (persistent micelles).^60,61^ Using the latter in soft templating, the pore size is expected to be locked to a constant value, whereas solvents enabling chain exchange (dynamic micelles) or even full micelle-core/solvent interactions (templating by BCP coassembly) might lead to fluctuating average pore sizes different from the value obtained by persistent micelle templating.^62^ With dynamic micelles as a soft template, the micelle and thus pore size deviate in both directions (up to 10–20% smaller or larger compared to the persistent micelle case^40,47,50,63^ ), which e.g., depends on the thermodynamic equilibrium size for a given (solvent) composition and temperature.^64–70^ If this thermodynamic situation changes (or is changed intentionally) during the soft templating process, then a dynamic micelle will adjust its size accordingly (as long as chain exchange is sufficiently fast). In contrast, a persistent micelle maintains a constant aggregation number.

**Figure 1:**
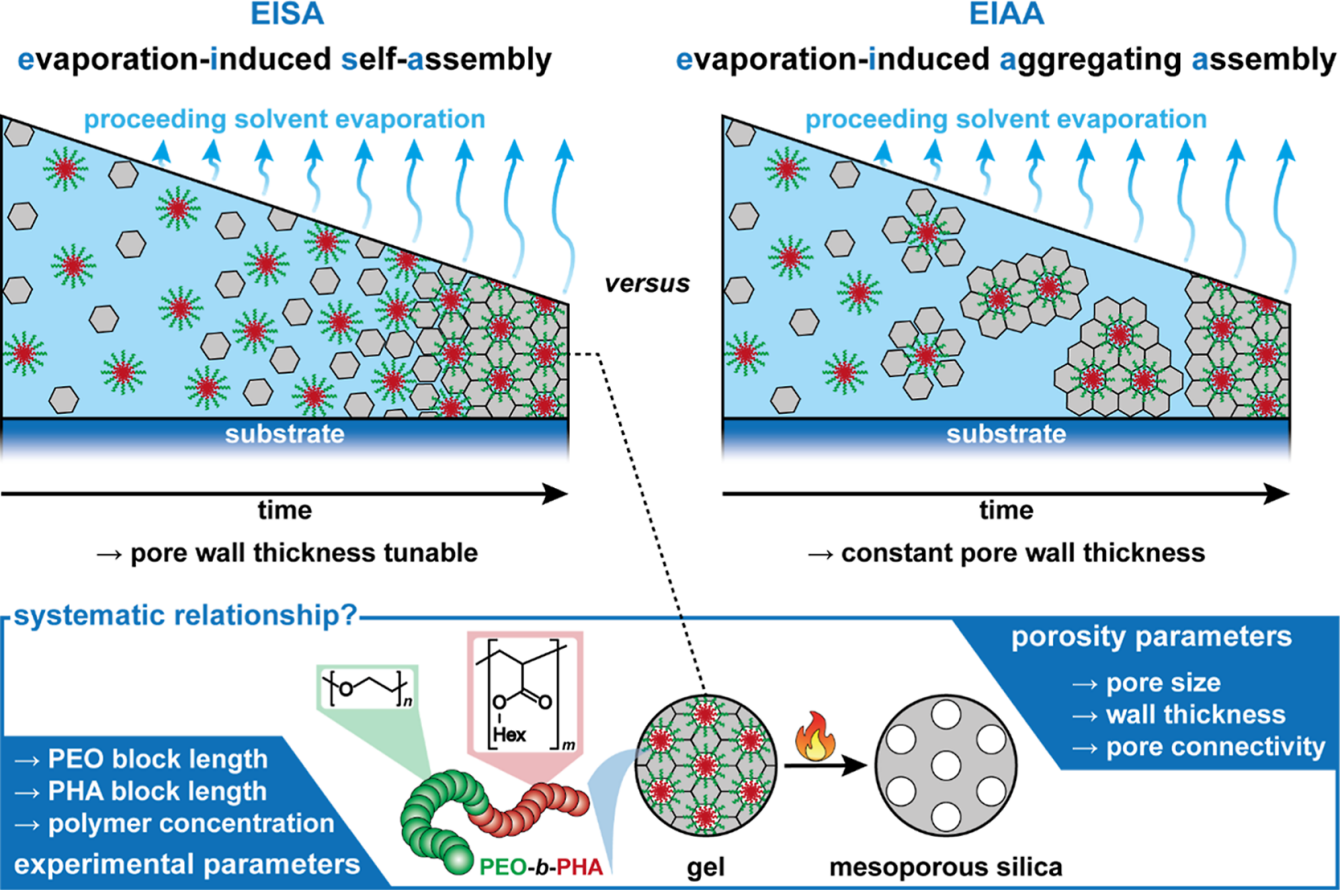
Schematic illustration of the EISA and EIAA mechanism for preparing mesoporous materials. In the case of EISA occurring for silica templated with PEO-*b*-PHA, the question arises how the experimental parameters (template composition and amount) are quantitatively related to structural parameters of the final mesoporous oxide.

Besides the micelle kinetics, also the polymer block constituting the micelle corona can affect the pore size: If solvation is not sufficient to fully embed the solvophilic block as a single chain inside the pore wall phase or the micelle core is enlarged featuring a larger core surface area, which needs to be stabilized, a fraction of the corona block will collapse on the micelle core leading to an increase in mesopore size.^71^

In this study, we thus tackle the role of (1) the block lengths of PEO-*b*-PHA copolymers and (2) the template amount in soft templating. Therefore, we prepared a library of 17 different samples of PEO-*b*-PHA comprising four different PEO block lengths and different PHA block lenghts, and employed them in the soft templating of silica. Characterizing the mesoporous powders by electron microscopy, nitrogen physisorption, and small-angle X-ray scattering (SAXS), we derive a quantitative relation between the individual block lengths and the pore size and reveal which of the synthesized PEO-*b*-PHA polymers can be used for generating spherical mesopores, and which pore size regime can be covered. This empirical relationship is then discussed in terms of fundamental principles of polymer physics. Soft templating with different amounts of PEO-*b*-PHA reveals the interplay of the micelle-to-micelle distance and the resulting pore size, wall thickness, and especially pore connectivity. The latter parameter was assessed by physisorption and especially by in order to unravel dependency on the PEO block length. In particular, this study intended to clarify to which extend the PEO block is a contributor to the micropore volume and possibly to the spherical mesopores and their size. Considering the similarity between soft-templated silica and zirconia in our previous study,^46^ the trends observed here likely can be generalized to other metal oxides and soft templates (as long as synthesis adjustments and precursor changes do not significantly hamper micellization), providing important guidelines for all kinds of systematic studies on porosity–property relationships and for the rational synthesis of adesired mesopore structure.

## Experimental Section

### Synthesis of the Macroinitiator PEO–Br

All block copolymers used throughout this work are based on four homopolymers of α-methyl-ω-hydroxy poly­(ethylene oxide) (PEO–OH) with an average molecular weight of 2 kDa, 5 kDa, 10 kDa, and 20 kDa, respectively, as provided by the supplier Sigma-Aldrich. A ^1^H NMR end group analysis gave a number-average molecular weight (*M*_n_) of the PEO–OH samples of 2.1 kDa (47 EO units), 6.0 kDa (137 EO units), 11.0 kDa (251 EO units), and 19.4 kDa (441 EO units), respectively.

Conversion of the PEO–OH homopolymers to the macroinitiators PEO–Br followed a Steglich esterification based on Lokupitiya et al.^48^ and reported in detail in our previous study.^46^ Briefly described, 1.0 equiv of PEO–OH (Sigma-Aldrich) was dissolved in a Schlenk round-bottom flask in anhydrous dichloromethane (DCM, 99.8%, Acros Organics) under argon by stirring at room temperature. Next, 1.2 equiv of 2-bromopropionic acid (99%, Acros Organics) were added with a syringe, followed by the addition of 0.4 equiv 4-dimethylaminopyridine (DMAP, ≥99%, Sigma-Aldrich) and 2.3 equiv of *N*,*N′*-dicyclohexylcarbodiimide (DCC, 99%, Sigma-Aldrich) during cooling with an ice bath. The turbid reaction mixture was stirred under an argon atmosphere at room temperature for 20 h. After excess DCC was quenched with 1 M hydrochloric acid (Grüssing GmbH), the suspension was diluted with DCM and filtered over a paper filter. The clear filtrate was concentrated under reduced pressure, and the resulting colorless residue was dissolved in tetrahydrofuran (THF, technical grade, Thermo Scientific, purified over basic aluminum oxide to remove peroxides). Precipitating twice in a 10-fold volume of cold diethyl ether (being cooled with an ice bath) and drying in a vacuum oven at 40 °C for 1 day delivered a colorless powder with a yield of 81% (20 kDa), 91% (10 kDa), 78% (5 kDa), and 89% (2 kDa).

In addition, a second PEO–OH sample (20 kDa according to Sigma-Aldrich, 16.4 kDa and 372 EO units according to NMR) was converted to PEO–Br with a yield of 88% as a reference for gel permeation chromatography (GPC) and dynamic light scattering (DLS) studies (see below).

### Synthesis of the PEO-*b*-PHA Copolymers

The PEO-*b*-PHA block copolymers were synthesized by a supplemental activator reducing agent atom transfer radical polymerization (SARA ATRP), as recently described.^46^ A copper wire being activated in a mixture of methanol and concentrated hydrochloric acid^72,73^ and wrapped around a stirring bar served as a reducing agent. In a typical reaction, 1.0 equiv of PEO–Br and 3.0 equiv of tris­(2-pyridylmethyl)­amine (TPMA, >98.0%, TCI CO.) were dissolved in anhydrous *N*,*N*-dimethylformamide (DMF, 99.8%, Acros Organics) by stirring at 40 °C in a Schlenk round-bottom flask in an inert atmosphere. After adding 1.5 equiv of CuBr_2_ (>99%, water-free, Acros Organics) under flowing argon, a freeze–pump–thaw cycle was carried out before injecting respective amounts (typically 10% more than the targeted degree of polymerization) of hexyl acrylate (98%, Sigma-Aldrich, purified from inhibitors by passing over basic aluminum oxide) into the solution. Following two further freeze–pump–thaw cycles, the copper-coated stirring bar was introduced under flowing argon, and the green solution was stirred at 70 °C under an argon atmosphere for 18 h. The obtained orange gel was diluted with THF and passed over a column of basic aluminum oxide. After removing the solvent with a rotary evaporator, the clear gel was dissolved in THF and precipitated in a 10-fold volume of cold methanol (cooled with an ethyl acetate/liquid nitrogen freezing mixture) twice. The colorless to yellow residue was transferred to a Petri dish and dried in a vacuum oven at 40 °C overnight. In the case of PEO_047_-Br-based copolymers, the precipitate was recovered by centrifugation at 5000 rpm for 5 min instead of filtration over a Büchner funnel. On average, around 60% of the expected mass (sum of macroinitiator and monomer) was obtained in the form of a colorless to yellow gel. All batches, including the amount of chemicals each, are listed in Table S1, while characterization by NMR and GPC is given in the Supporting Information.

### Synthesis of Mesoporous SiO_2_ Powders

Mesoporous SiO_2_ powders were prepared by soft templating in analogy to our past article.^46^ In this sol–gel synthesis, relying on the studies of Weller et al.^74^ and Cop et al.,^75^ the corresponding amount of soft template (see Table S2)—namely, PEO-*b*-PHA or PIB_50_-*b*-PEO_45_ (BASF)^27,75–78^ —was dissolved by ultrasonication at 37 kHz and 40 °C in 1 mL of absolute ethanol (99.8%, Fisher Chemical) within 40 min. Meanwhile, 130 μL of tetraethyl orthosilicate (TEOS, 98%, Sigma-Aldrich) was dissolved in 0.5 mL absolute ethanol by stirring at room temperature in a 5 mL PTFE cup. Both the clear polymer solution and 40 μL of deionized water were added to the metal oxide precursor. After 5 min of stirring, 10 μL of a concentrated hydrochloric acid (37 wt %) were added prior to stirring for 1 h at room temperature. The clear solution was kept at 40 °C under a glass dome for 2 days, followed by a second drying step in a vacuum oven at 40 °C for 1 day. The colorless gel was calcined at 350 °C for 1 h to remove the soft template, and at 500 °C for 4 h to obtain the final metal oxide (heating ramp each: 2 K min^–1^), resulting in *circa* 35 mg of a colorless powder after grinding in an agate mortar.

### Characterization Techniques

Proton-nuclear magnetic resonance (^1^H NMR) spectra were acquired in CDCl_3_ at 25 °C with Bruker AVANCE II 400 MHz and Bruker AVANCE III 400 MHz HD as well as a Bruker Ascend AVANCE 4 Neo 7 700 MHz spectrometer and evaluated with MestReNova 14.1.2. All signals were referenced to the solvent signal at δ = 7.26 ppm. GPC with simultaneous UV (TSP UV 1000) and differential refractive index (Shodex RI-101) detection was executed at room temperature using tetrahydrofuran as an eluent at a flow rate of 0.5 mL min^–1^. The stationary phase consisted of a 300 mm × 8 mm PSS SDV linear M column packed with 3 μm particles (molecular weight range of 1 × 10^2^ to 1 × 10^6^ Da). Prior to the injection of a 100 μL sample solution containing around 0.15 wt % polymeric sample, it was filtered through 0.45 μm filters. Poly­(styrene) standards (PSS, Mainz, Germany) were used for calibration. DLS experiments were performed on a Zetasizer Nano series instrument from Malvern. For each measurement, sample solutions with around 1 mg mL^–1^ polymer in ethanol and methanol, respectively, were characterized in three runs of 13 cycles. The data were exported using the corresponding application, Zetasizer Software.

Nitrogen physisorption experiments were carried out at 77 K on a Quadrasorb evo instrument (Quantachrome Instruments, Boynton Beach, FL) after sample degassing at 200 °C for 6 h in order to remove attached water and gases. The data obtained were analyzed with the aid of the software ASiQwin by applying a nonlocal density functional theory (NLDFT) method dedicated to nitrogen at 77 K on siliceous/oxidic materials, assuming a cylindrical pore geometry. The pore size distribution was obtained from the adsorption branch by applying a dedicated metastable adsorption branch kernel, which correctly takes into account the delay in pore condensation due to the metastable pore fluid.^2^

Scanning electron microscopy (SEM) images were obtained with a Zeiss GeminiSEM 560 microscope using an InLens detector, a working distance of 2.5 mm, and an acceleration voltage of 1 kV. To enhance the conductivity, samples were sputtered with platinum using a Leica EM ACE600 sputter coater. All images were evaluated with Fiji ImageJ software.

SAXS measurements were carried out by the laboratory SAXS instrument SAXSpoint 2.0 by Anton Paar using point-focused (spot size of 500 μm) and slit-collimated Cu Kα radiation (λ = 0.1541 nm) from a microsource operating at 50 W and a Dectris EIGER2 R 1 M hybrid pixel area X-ray detector. Powder samples were placed into an Anton Paar solid sample holder comprising a 1 mm thick metal plate with 20 square holes mounted on a motorized X/Y-stage. The sample plate was sealed at both sides with a vacuum-tight sealing tape. SAXS images of the samples and the background (sealing tapes) were recorded in vacuum (around 1 mbar and 25 °C) at a sample-to-detector distance of 575.65 mm. For each sample and background, respectively, 15 single images with 2 min exposure time each were recorded, averaged, and radially integrated in order to obtain the 1D-SAXS curves. The scattering curves were fitted using SASfit 0.94.11 with a model consisting of a form factor for spheres with a Gaussian size distribution and a lattice factor applying the implemented decoupling approximation.

Scanning transmission electron microscopy (STEM)-based tomography was executed with a Thermofisher Scientific Themis 300 transmission electron microscope equipped with probe aberration correction and operated at an acceleration voltage of 300 kV. The silica powder (around 10 mg) was ground, suspended in ethanol, and dropped on a 100 × 400 mesh carbon-coated copper grid purchased from Quantifoil Micro Tools GmbH. Gold nanoparticles (*d*_Au‑np_ = 12 nm) were deposited onto the sample as fiducial markers for image alignment. The grid was cleaned twice for 30 s using a Fischione 1070 plasma cleaner in an argon–oxygen atmosphere with a power of 50%. A high-angle annular dark-field (HAADF)-STEM tilt series was acquired over a tilt range of −72° to 70° (step size: 2°) and with a pixel size of 0.81 nm using the Xplore3D software (Thermofisher Scientific) with auto focus and tracking before acquisition. A small convergence angle of roughly 8.5 mrad was used to increase the depth of focus. With the aid of the gold nanoparticles, image alignment was done in IMOD Version 4.11.7 (University of Colorado) with a residual alignment error of 0.606 nm (0.374 px). The 3D reconstruction (with a voxel size of (1.62 nm)^3^) was obtained from the aligned tilt series with the simultaneous iterative reconstruction technique and 100 iterations in Inspect 3D 4.4 (Thermofisher Scientific). Denoising with a median filter in ImageJ and binarization by global thresholding in Avizo 2021.1 (Thermofisher Scientific) of the tomograms yielded an initial segmentation. The segmented volume was purified by the removal of unconnected islands smaller than 15 voxels, which originate from reconstruction artifacts. Skeletonization of a cropped region of the image stack was executed, as reported by Cheng et al.^79^ using ToolIP with MAVIkit 2024 (Fraunhofer ITWM) in order to elucidate the local pore connectivity. Pore and wall size distributions were obtained with a local thickness evaluation using the corresponding plugin in ImageJ after subtracting the background with a Python code applying a rolling ball radius of 30 pixels (48.6 nm) as recently described by us.^80^ While the experimental values of tilt range, unbinned pixel size, residual alignment error, voxel size of the 3D reconstruction, and the rolling ball radius given here correspond to the 85 vol % sample, the experimental details of the remaining samples (69 and 75 vol %) are listed in Table S3. Thermogravimetric studies coupled with mass spectrometry (TG-MS) were carried out in synthetic air using a STA40PC thermoscale provided by Netzsch within a temperature range from 30 to 1000 °C with a heating ramp of 2 K min^–1^ and the heating ramp used for the silica synthesis, respectively. Meanwhile, a mass spectrum covering an *m*/*z* interval between 12 and 100 was recorded constantly with the aid of a QMG451 quadrupole mass spectrometer by Balzers.

## Results and Discussion

### PEO-*b*-PHA Block Copolymers

The synthesis of PEO-*b*-PHA block copolymers featuring a hydrophilic poly­(ethylene oxide) and a hydrophobic poly­(hexyl acrylate) block follows a two-step protocol described in our last article and summarized in Scheme S1: Steglich esterification yields the PEO–Br macroinitiator, to which the PHA block is attached by supplemental activator reducing agent atom transfer radical polymerization (SARA ATRP).^46^ Studying the influence of both the PEO and the PHA block lengths on the pore size after soft templating requires a systematic matrix of block copolymers with tailored block lengths. Thus, four different PEO–Br macroinitiators were prepared starting from PEO homopolymers with average molecular weights of 20, 10, 5, and 2 kDa (as reported by the supplier). According to NMR end group analysis,^46^ the degree of polymerization of the macroinitiators amounts to 441, 251, 137, and 47 EO units, respectively (see ^1^H NMR spectra in Figures S1 and S2). From these PEO_*n*_–Br macroinitiators, 18 different PEO_*n*_-*b*-PHA_*m*_ samples were prepared. According to NMR (Figures S1 and S2), the degrees of polymerization *n* and *m* of the PEO and PHA block, respectively, are obtained as plotted in Figure 2 and Table S4.

**Figure 2:**
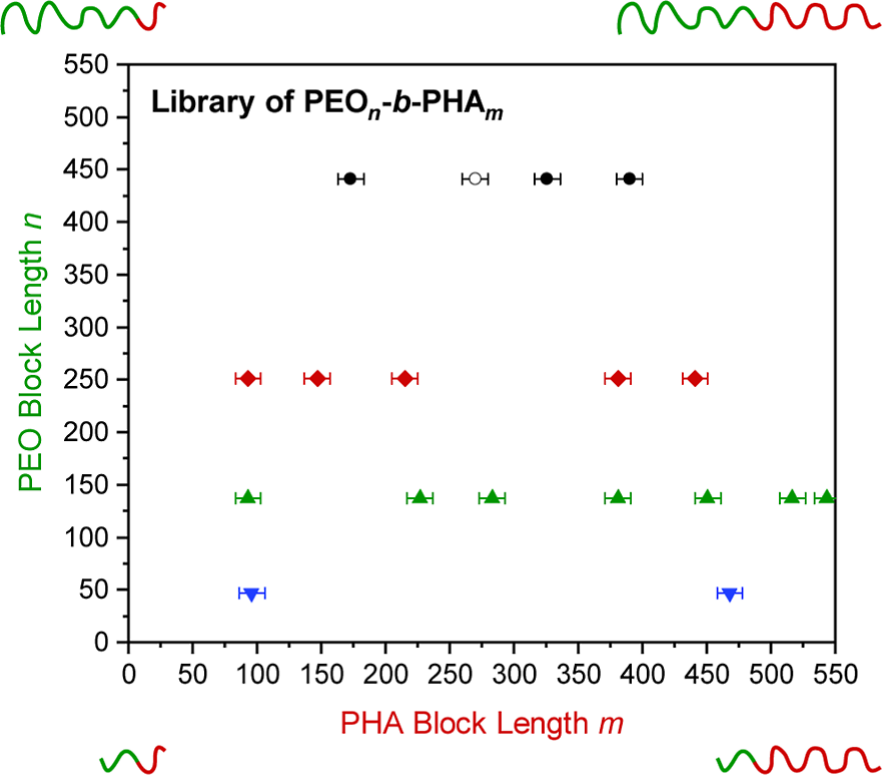
Library of PEO-*b*-PHA copolymers synthesized from PEO–Br macroinitiators with a PEO block length of 441 (black), 251 (red), 137 (green), and 47 EO units (blue). Sample PEO_441_-*b*-PHA_270_ (open symbol) was neglected for pore size evaluation due to the high polydispersity.

The PEO-*b*-PHA samples possess a polydispersity index of around 1.4 (Table S4), similar to the copolymers in our previous study.^46^ In general, the GPC curves (Figure S3A) are consecutively shifted to smaller elution volumes with increasing NMR-based PHA block length, as expected from the increasing molecular weight and thus polymer size. The only exception is sample PEO_441_-*b*-PHA_270_ showing an almost identical GPC trace as polymer PEO_441_-*b*-PHA_326_ but, in addition, a higher amount of residual PEO homopolymer (shoulder at high elution volumes).^81^ As a result, both the GPC-based average molecular weight and especially the NMR-based PHA block length are underestimated because both methods rely on the average over the entire sample. Hence, sample PEO_441_-*b*-PHA_270_ was excluded from quantitative studies on the dependency of the pore size on the block lengths and only used for concentration-dependent investigations, in which the relative pore size evolution is of importance. Due to calibration to poly­(styrene) (PS), GPC is not capable of providing absolute weight distributions. However, a plot of the apparent number-weighted molecular weight from GPC against the NMR-based degree of polymerization of the PHA block still yields a linear trend. Indeed, the plots in Figure S3B confirm that all samples follow such a linear behavior with a slope of around 100 Da. Taking the molar mass of the repeating unit in the PHA block of 156 Da into account, we find that the order of magnitude is reasonable. Both the linear trend and the reasonable slope thus support that the block lengths from NMR (Figure 2) are reliable.

The soft templating process is based on the ability of PEO-*b*-PHA to self-assemble into spherical micelles, being converted to spherical mesopores upon calcination. In this regard, the size of the block copolymers in solution governs the pore size and is of particular relevance. However, our previous DLS study^46^ showed that PEO-*b*-PHA does not form micelles in ethanol—the solvent used for soft templating—but dissolves as Gaussian coils. Yet, such a study using DLS (Figure 3A) is valuable to back up the block lengths obtained from NMR, as the coil size should depend on the length of both blocks. The reason why PEO-*b*-PHA still forms micelles upon solvent evaporation^46^ in the soft templating of silica can be rationalized by the solubility of the respective polymer blocks estimated by the Hildebrand solubility parameters *δ*.^82–84^ During the evaporation of ethanol (*δ* = 26.2 √MPa), the reaction mixture is enriched with the silica precursor TEOS (δ = 14.6 √MPa, calculated with an enthalpy of vaporization of 50 kJ mol^–1^) leading to a decrease in δ of the solution and approaching the solubility of the hydrophobic PHA block (*δ* = 16.6 √MPa) and thus the micellization regime.^48^ Following the DLS study in our previous work, micellization occurs, starting from a TEOS fraction of 80 vol %, which equals a *δ* = 16.9 √MPa (upon volumetric averaging), being close to the PHA solubility. Regarding the solubility of the PEO block (*δ* = 20.3 √MPa),^85^ PEO-*b*-PHA might possibly even form inverse micelles in the highly TEOS-enriched low-*δ* regime, being close to PHA but far away from the PEO solubility. This scenario, however, is unlikely because the (partial) hydrolysis of TEOS already occurs due to environmental humidity (to a small extent) and particularly during the synthesis due to the added water, which elevates the actual solubility parameter of the silica precursor. Also, it should be noted that the added amounts of water and hydrochloric acid further increase *δ* of the sol–gel mixture (away from the dynamic micelle regime), but a meaningful estimation is difficult due to the unknown amount of water removed by coevaporation with ethanol, hydrolysis of TEOS, and absorption through the silica gel.^86^ Therefore, a clear statement of whether the micelles are persistent or dynamic is impossible for our synthesis. Yet, the extreme case of polymer coassembly (bulk behavior with the least solvent selectivity)^62,86^ can be excluded since micellization in (TEOS-enriched) solution within an EISA mechanism was identified (prior to gelation) by cryo-electron microscopy and SAXS in solution in our previous study.^46^ In contrast to ethanol, PEO-*b*-PHA showed micellization in methanol,^22,50^ giving access to evaluate the micelle size in dependency on the PEO and PHA block length (Figure 3B).

**Figure 3:**
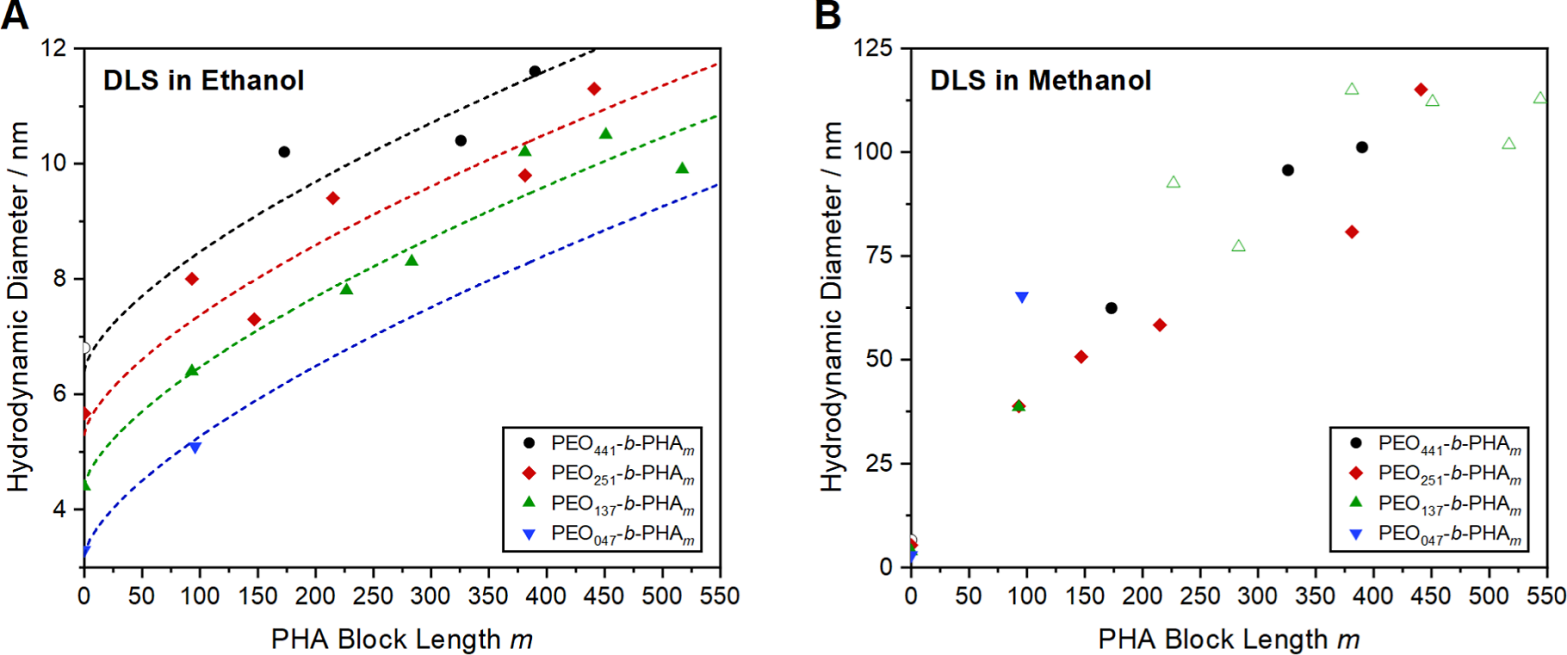
Hydrodynamic diameter from DLS of PEO-*b*-PHA copolymers of varying PEO block lengths (441 EO units in black, 251 in red, 137 in green, and 47 in blue) dissolved in (A) ethanol and (B) methanol. Entities expected to be vesicles instead of micelles (see SEM images below) are shown with open symbols. Due to a lack of sufficient amounts of PEO_441_–Br, PEO_372_–Br (open black circle) was investigated for comparison.

The DLS data of the polymers dissolved in ethanol (Figures S4 and S5) clearly show an increase in hydrodynamic diameter with increasing PHA block length, but also with increasing PEO block length. This observation becomes more apparent in a plot of the average hydrodynamic diameter against the PHA block length *m*. In the case of all PEO block lengths, Figure 3A reveals a degressive increase of the coil size with increasing PHA block length following roughly an *m* ^0.67^ dependency. Despite the uncertainties of these measurements (ca. ± 1 nm) and the pronounced scattering of the data points in the case of large PEO blocks, the exponent in the power law (0.67) is close to Flory’s exponent of 0.6 observed for dissolved PEO by DLS^87^ and hints at a coiled state. Furthermore, the increase of the hydrodynamic diameter with increasing PEO block length can be clearly envisioned in Figure 3A, yielding a gradual shift of the fit curves toward higher diameters. So, the observed trends for the PEO and PHA block length variation further support the NMR-based degrees of polymerization.

Going to methanol, the entities observed by DLS are significantly larger than those in ethanol (Figures S4 and S5), except for the homopolymers (*m* = 0), whose hydrodynamic diameter remains the same as in ethanol. This observation confirms the self-assembly of PEO-*b*-PHA copolymers in methanol. Regarding a quantitative evaluation, an accurate scaling law is difficult to derive, as the data points are severely scattered (Figure 3B). However, the hydrodynamic diameter appears to scale linearly with the PHA block length (*m* ^1^), which is reasonable for micelles^71,88,89^ although the exact exponent depends on the extent of segregation of both blocks.^90^ Especially, if those block copolymer molecules are neglected that form vesicles instead of micelles (open symbols in Figure 3B) according to soft templating (see SEM evaluation below), a linear trend becomes obvious with a slope of roughly 20 nm per 100 HA units. A comparable slope (*circa* 25 nm per 100 S units) is found if the DLS-based diameter of PEO-*b*-PS micelles is plotted against the PS block length,^89^ which strengthens the trend observed here. On the other hand, the influence of the PEO block length on the hydrodynamic diameter becomes less apparent, although the PEO corona is expected to have a strong contribution to the micelle size as well.^91^ If sample PEO_251_-*b*-PHA_441_ is regarded as an outlier (e.g., if the observed entity is a vesicle), a size difference between PEO_441_-*b*-PHA_*m*_ and PEO_251_-*b*-PHA_*m*_ samples can be observed, although it is rather small. However, the degree of polymerization influences not only the micelle size but also the aggregation number,^88,90^ i.e., the number of chains building the micelle. Thus, a possible change of the aggregation number upon varying the block length might compensate for the different PEO block lengths, annihilating a PEO dependency. This becomes even more complicated since the existence of a micelle equilibrium state (which can be achieved by chain exchange of dynamic micelles or promoted by sonication as performed here) or a kinetic trapping of the micelles (persistent micelles) here remains unclear from DLS alone as the presence of single chains in solution (hinting at dynamic micelles) might be overshadowed by the larger micelles possessing a higher scattering intensity than the smaller unimers.^50,59,61,64,92^ Furthermore, the high sensitivity of micellization and micelle size to solvent and cosolvent effects, in which trace water/solvents in the polymer samples might alter the polarity of the medium, can cause a certain variation of the micelle size.^50^ A deeper evaluation requires scattering experiments, but is not the focus of this work. Moreover, the DLS study still suggests that the micelle size can be tailored continuously over a broad range by adjusting (particularly) the PHA block length. Hence, the polymer library prepared here represents a suitable basis for a soft templating study.

### Influence of the Block Lengths on the Pore Size

To understand the individual impact of the PEO and PHA block length on the pore size, the presented block copolymers were applied for soft templating of silica according to a protocol we previously reported.^46^ Although direct micellization takes place in methanol (Figure 3B), we kept using ethanol as the solvent for soft templating here due to the higher solubility of PEO-*b*-PHA copolymers in ethanol compared to methanol to avoid solubility limitations upon solvent evaporation (especially during the concentration series in the next section). The benefit of mesoporous silica as a model material lies in a facile synthesis resulting in an almost ideal replica of the lyotropic phase.^93–97^ In addition, we already characterized the pore structure of PEO-*b*-PHA-derived mesoporous silica in depth^46^ simplifying the pore size evaluation here. Using the block copolymers in Figure 1 as soft templates in a sol–gel-based synthesis, the respective mesoporous silica powders in Figure 4 are obtained.

**Figure 4:**
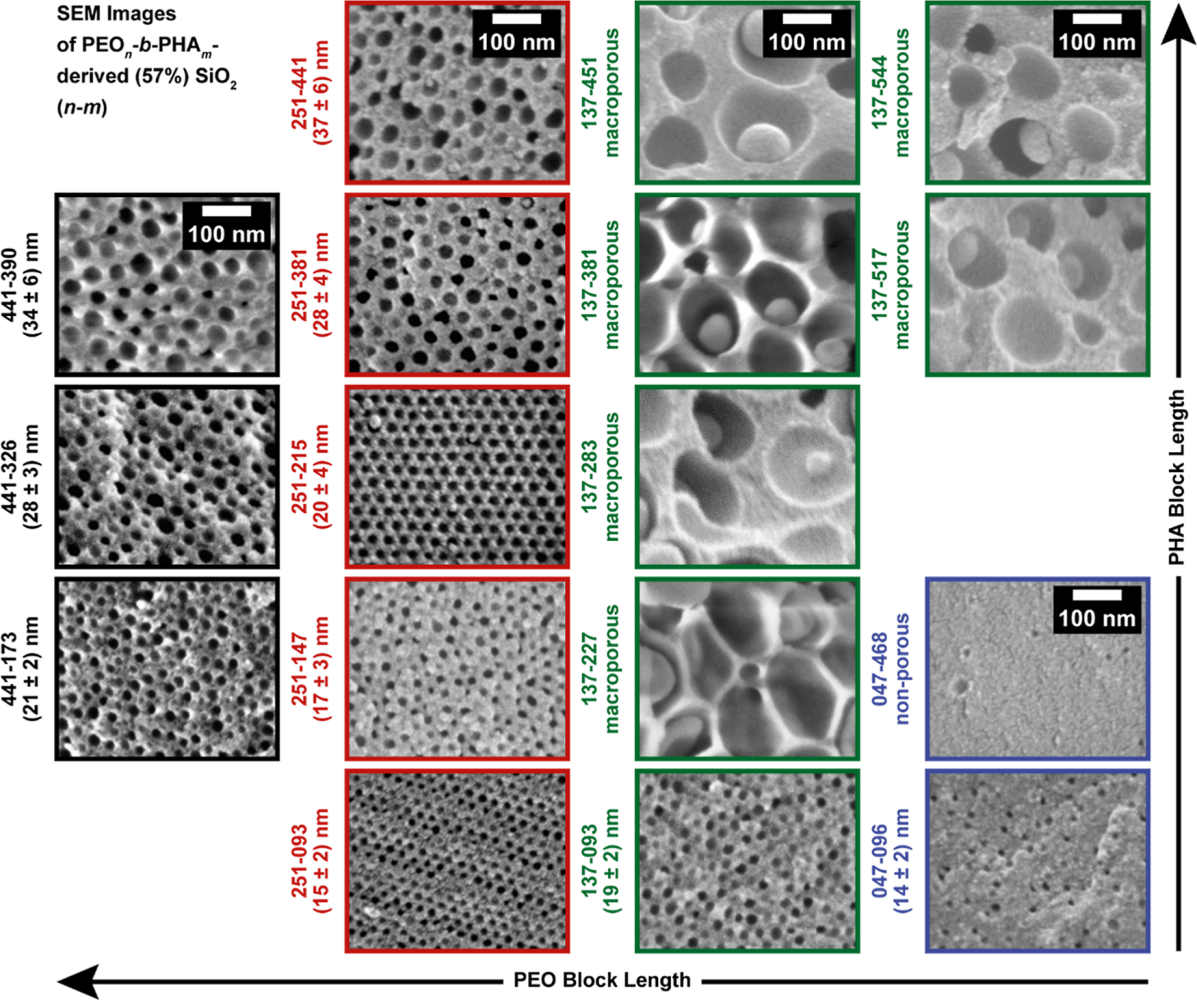
SEM images of mesoporous silica prepared with 57 vol % PEO_*n*_-*b*-PHA_*m*_ with a PEO block length of *n* = 441 (black), 251 (red), 137 (green), and 47 (blue), while the PHA block length *m* increases from the bottom to the top for each PEO block length. The mesopores are spherical in shape, as confirmed by SAXS and electron tomography.

From SEM, it becomes obvious that not all PEO-*b*-PHA samples are able to produce mesoporous silica with spherical pore geometry. In fair alignment with our last study^46^ and the concept of packing parameter,^98,99^ a sufficiently large PEO block is necessary to ensure a stable spherical micelle. With decreasing PEO block length, i.e., increasing volume fraction of the PHA core, lamellae are preferred over spheres, leading to a vesicular structure. Further decrease in the PEO volume fraction theoretically results in the formation of an inverse micelle, which is, however, not stable in the hydrophilic medium of this sol–gel reaction. Consequently, a transition from micelles to vesicles occurs in PEO_137_-*b*-PHA_*m*_ with *m* > 100, yielding macropores with nanoparticles in their center (from encapsulating hydrophilic precursor solution in the vesicle), while in the boundary case PEO_047_-*b*-PHA_468_ almost no pores are found (Figure 4). All remaining silica samples possess an ordered mesoporous structure with spherical pores, which hints at the micellization of the underlying amphiphilic BCP templates. Silica templated with PEO_047_-*b*-PHA_096_ represents rather a border case: Although several small mesopores are visible in the SEM image, their number appears to be rather small. Also, the nitrogen physisorption isotherm of this sample (Figure S6) reveals a majorly microporous sample. Both methods evoke the hypothesis that this BCP is at the borderline of forming stable micelles. Overall, we find that nitrogen physisorption confirms the observation from SEM, as mesoporous samples templated with micelles can be easily distinguished from those in which vesicles were present during the soft template process. The physisorption isotherms in Figure S6, which correspond to mesoporous samples (soft templates with high PEO volume fraction), possess a plateau at high relative pressures, whereas the macroporous samples (soft templates with low PEO volume fraction expected to form vesicles) do not reach such a plateau. This becomes particularly apparent in the desorption branch at high relative pressures. SAXS of the silica samples provides analogous results (Figure S8), yielding a SAXS curve with form factor oscillations in the case of mesoporous samples, a SAXS curve with a slow decay in the case of macroporous ones, and a SAXS curve with a steep decay in the case of nonporous ones.

Regarding quantitative pore size evaluation, all samples were characterized by SEM, nitrogen physisorption, and SAXS. In this regard, the same approach as in our last study^46^ is used. Briefly explained, mesopore sizes from SEM were obtained by averaging a statistical sample of 50 mesopores. In the case of nitrogen physisorption, an NLDFT method [N_2_ at 77 K on silica (cylindrical pores, NLDFT, adsorption branch) of the ASiQwin software], assuming cylindrical pore geometry, is applied on the adsorption branch of the nitrogen isotherms (Figure S6), which results in the pore size distributions in Figure S7. This NLDFT method correctly considers the delayed condensation due to the nucleation barrier of the vapor–liquid transition^2^ but requires a correction to take the actual pore geometry into account. Multiplication with a geometrical correction factor of 1.35 for conversion of the cylindrical to a spherical pore geometry^6^ provides the final mode pore size, which proved to be meaningful.^46^ This approach is necessary because the nitrogen NLDFT kernel for spherical pores is not programmed yet for pores larger than 30 nm. SAXS curves were modeled with a theoretical curve using the Percus–Yevick lattice factor^46,100–103^ and a form factor for polydisperse spheres, giving access to a mean pore size. Since all three methods provide comparable results (see Table S5), the following quantitative relations are based on SEM-derived pore sizes, as the SEM evaluation is straightforward, meaningful, and robust for a wide range of pore diameters. Despite being only a local method, the entire pore size regime can be covered, while physisorption and SAXS become inaccurate for large mesopores. Above a pore diameter of 40 nm, pore condensation in the isotherm is very closely shifted to a relative pressure of *p p*_0_^–1^ = 1, so that the pore size distribution of our materials reaches the upper limit of detection and provides the same value, although the materials are different in pore size. Similarly, the form factor minimum in SAXS approaches the lower limit of detection of the scattering vector for pore diameters above 35–40 nm. In combination with the increasing polydispersity causing less pronounced form factor oscillation, an accurate pore size determination becomes challenging.

From a qualitative point of view, an increase in mesopore diameter with increasing PHA block length is clearly visible by SEM (Figure 4), physisorption (Figure S7), and SAXS (Figure S8) analysis, while the PEO block length appears to have only a minor influence. A quantitative consideration confirms this observation. As shown in Figure 5, a plot of the mesopore diameter against the PHA block length yields a common linear relation for all PEO block lengths. The pore diameter follows, in good agreement, the trend observed for the micelle size (Figure 3B), although being less scattered and being only around 30% of the micelle diameter on average. Within this set of PEO-*b*-PHA soft templates, a pore size range of roughly 15 to 40 nm can be covered, where empirically, the pore size *d*_p_ depends linearly on the PHA block length *m* according to eq 1.

1 $$d_{p}\left(57\%\right)=0.06\;nm\cdot m+9.9\;nm$$

**Figure 5:**
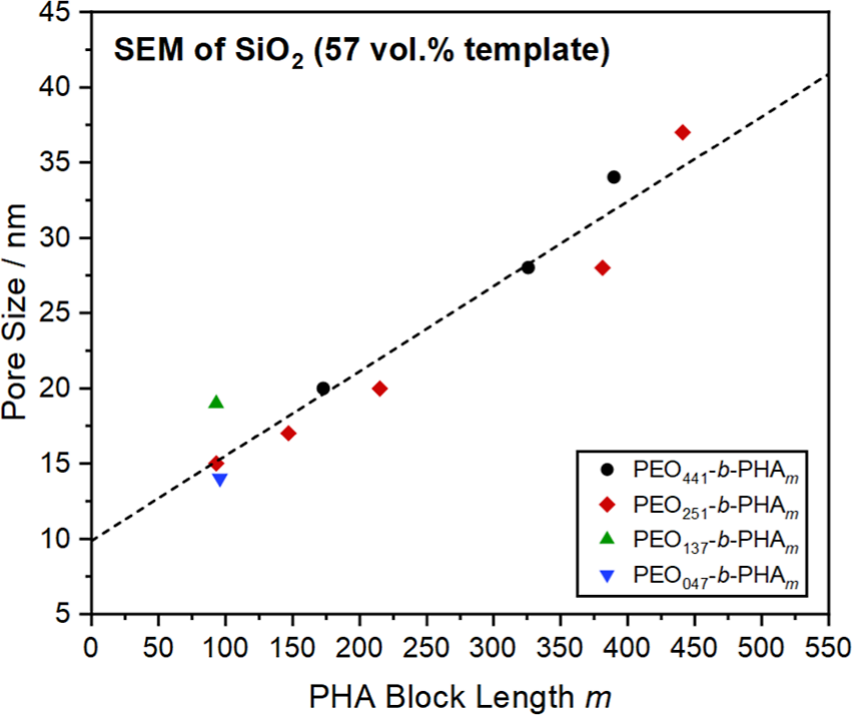
SEM-based pore size of mesoporous silica prepared with 57 vol % PEO_*n*_-*b*-PHA_*m*_ with a PEO block length of *n* = 441 (black), 251 (red), 137 (green), and 47 (blue) plotted against the PHA block length from NMR.

Note that a block length above 500 HA units is challenging to achieve (in particular, for large PEO blocks) due to the high viscosity of the BCP during polymerization. Although the synthesis of mesoporous silica here might be accomplished through dynamic micelle templating, the observed relation shows a good agreement with the pore size evolution as a function of the PHA block length achieved with persistent PEO-*b*-PHA micelles by Stefik and co-workers (see Figure S9, note that the kind of metal oxides differ).^62^ Yet, as presented there, it is important to mention that the synthesis history (stirring time, sonication, etc.) can influence the pore size as well, and thus an assignment of the targeted pore size to the polymer block length is strictly valid only for one synthesis protocol. Furthermore, it is worth mentioning that the pore size is dependent on the applied BCP concentration,^46^ which will be discussed below in more detail.

The absence of a dependency on the PEO block length is surprising if studies on other soft templates like *n*-alkyl-PEO^71^ are considered, in which the PEO block has a similarly large influence on the pore size as the hydrophobic block. Assuming a “three-phase model”,^71,90,104^ in which a fraction of the PEO block collapses on the PHA micelle core, as suggested in our last study,^46^ a possible explanation can be found: A certain amount of the PEO block collapses onto the PHA core, while the remaining fraction is part of the hairy micelle corona penetrating the silica wall and ending up as a micropore channel. As a result, excess PEO does not contribute to the mesopore size but to the micropore volume (see the discussion in the last section). Thus, all mesopores experience a constant contribution of the collapsed PEO of around 10 nm to each mesopore (offset of regression function in Figure 5), irrespective of the PEO-*b*-PHA soft template. Billet et al. observed a similar behavior for titania soft-templated with block copolymers composed of poly­(*N*,*N*-dimethylacrylamide) (PDMA) and PS with two different PDMA block lengths and a tailored PS block length.^105^ Independent of the PDMA block length, all copolymer samples introduced mesopores, whose size depends only on the PS block length. Regarding the slope stating a pore size increase of 6 nm per 100 HA units, a comparison with studies on PEO-*b*-PS of Zhu et al. is possible. They observed a pore size increase of around 3 nm per 100 S units in mesoporous tungsten oxide,^106^ which is of a similar order of magnitude to our findings, although the slope most likely depends on the size of the repeating unit and hence might be a parameter being specific for each polymer family.^16,71,105,106^ A direct correlation of the slope with the size of the repeating unit is difficult, however, due to the unknown extent of stretching of the PHA chain in the micelle core and the mesopore shrinkage during calcination. All in all, PEO-*b*-PHA demonstrates a clear pore size evolution as a function of PHA block length, enabling a tailor-made pore size between 15 and 40 nm.

### Influence of the Template Concentration on the Porosity

Next to the size of mesopores, the pore wall thickness features a second important structural parameter of mesoporous materials. Normally, an adjustment of the template concentration, i.e., the ratio of soft template to precursor in the soft templating process, should enable tuning of the pore wall thickness. However, while Lokupitiya et al. proposed a deliberate tailoring of the pore wall thickness upon maintaining a constant pore size by persistent micelle templating,^48^ our previous study hinted at a more complex influence of the template concentration on the porosity.^46^ To investigate this topic in more detail, we templated silica in a case study with PEO_441_-*b*-PHA_270_ over a broad range of template concentration (Figure 6). The soft template concentration is given here as polymer volume fraction Φ (in vol %) describing the ratio of BCP volume *V*_BCP_ to total volume (polymer volume and silica volume *V*_oxide_), as shown in eq 2 ^107^

2 $$\Phi =\frac{V_{BCP}}{V_{BCP}+V_{oxide}}$$

**Figure 6:**
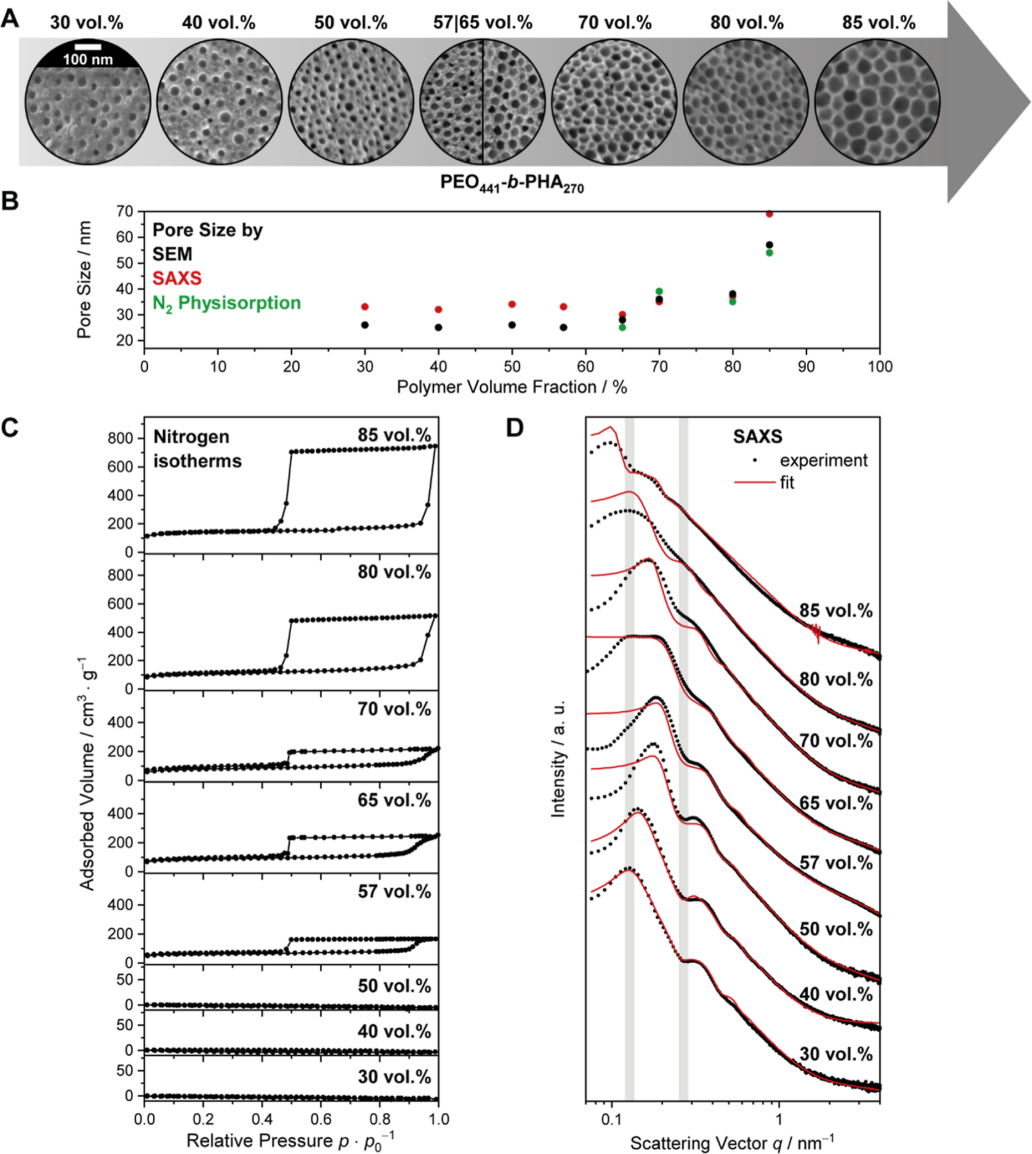
(A) SEM images, (B) nitrogen isotherms, and (C) SAXS curves of mesoporous silica prepared with different amounts of PEO_441_-*b*-PHA_270,_ as well as (B) the pore size from SEM (black), physisorption (green), and SAXS (red), which depends on the applied polymer amount.

The volume of silica *V*_oxide_ is obtained by converting the amount of precursor to a molar amount of silica under the assumption of the full conversion of tetraethyl orthosilicate to silicon dioxide. This assumption seems reasonable since the theoretical mass of 35 mg silica per batch was obtained experimentally in most samples. Molar mass and density of amorphous silica (2.2 g mL^–1^) then yield the desired volume. The volume of PEO-*b*-PHA *V*_BCP_ is generated by the division of applied polymer mass by its density ρ_BCP_. The latter is estimated according to eq 3 by dividing the number-weighted molar mass from NMR *M*_n_ by the sum of the blocks’ volumes (with the number of EO and HA repeating units *n* and *m*, their mass *M*_EO_ = 44 g mol^–1^ and *M*_HA_ = 156 g mol^–1^, and the density^48^ of the polymer blocks ρ_PEO_ = 1.13 g mL^–1^ and ρ_PHA_ = 1.065 g mL^–1^, respectively)

3 $$\rho_{BCP}=\frac{M_{n}}{\frac{nM_{EO}}{\rho_{PEO}}+\frac{mM_{HA}}{\rho_{PHA}}}$$

The polymer volume fraction Φ according to eq 3 thus describes the theoretical porosity of the final mesoporous metal oxide. A comparison with the experimental porosity from nitrogen physisorption (Figure S10G) shows that this descriptor is meaningful, possessing a similar evolution, although a constant overestimation of about 15% is observed. A reasonable explanation represents the fact that the BCP most likely is coiled more densely in the micelle state than in the homopolymer. Consequently, the density should be higher than that in a homopolymer assumed for calculation in eq 3.

The SEM images (Figure 6A) confirm qualitatively the presence of ordered spherical mesopores over the entire concentration range, as well as a decreasing pore-to-pore distance and a pore size increase upon increasing the polymer volume fraction. Quantitatively, a plot of the pore size against the applied polymer amount (Figure 6B) reveals a more complex dependence of the pore size on the polymer volume fraction: Initially, the pore size remains the same, but when a threshold of 57 vol % is exceeded, the mesopores start to grow steadily. At 85 vol %, the pore size is twice as large as in the initial constant region.

The SAXS curves in Figure 6D underline these results and show that the form factor minimum at *q* values of around 0.25 nm^–1^ (being mostly affected by the pore size) remains the same at low polymer volume fractions (30–65 vol %), implying a constant pore size. Simultaneously, the lattice factor maximum (0.1–0.2 nm^–1^), being strongly connected to the pore-to-pore distance, shifts steadily toward higher *q* values. This trend clearly demonstrates a decreasing pore-to-pore distance and thus wall thickness upon increasing the soft template concentration and confirms that the templating procedure here must follow an EISA mechanism. Otherwise, such a continual pore wall adjustment would hardly be possible. Above 65 vol %, both form factor minimum and lattice factor maximum shift continuously toward lower *q* values as a result of the increasing pore size. Note that an increasing pore size leads to an increase in pore-to-pore distance as well if a wall size decrease cannot compensate for or outweigh the pore size increase. On a quantitative level, the pore size from SAXS (Figure 6B) is in good agreement with that from SEM, especially considering the uncertainty of SAXS (mostly affected by the polydispersity of the pore size) of up to 10 nm and SEM (±5 nm), which are omitted in Figure 6B for clarity but are given in Figure S10A.

Nitrogen physisorption of these samples provides insights into the underlying mechanism causing this complex pore size evolution. At low polymer volume fractions, isotherms of apparently nonporous silica are observed. However, both SEM and SAXS clearly confirm the presence of ordered mesopores in the first three samples. Only from 57 vol % onward nitrogen isotherms are obtained, exhibiting the expected hysteresis loop. These results indicate that the silica powders prepared with 30–50 vol % PEO_441_-*b*-PHA_270_ are mesoporous, but the pores are not accessible for nitrogen. The polymer concentration at which the pore system becomes accessible according to physisorption coincides with the point from which on the pore size starts to increase (57–65 vol %). Together with the findings of our previous study on PEO collapse,^46^ the following mechanism can be proposed: At low soft template amounts, the hairy micelles in the gel are evenly distributed within the inorganic gel but too far apart to create an interconnected pore network. Upon increasing the amount of the soft template, the micelles approach each other until they are sufficiently close so that the PEO chains of the micelle corona penetrating the inorganic matrix can generate continuous micropore channels, which ensure an accessible mesopore space. Further increasing the soft template concentration forces the micelles to interact, leading to a collapse of PEO onto the PHA core to gradually increasing extents, which increases the resulting pore size. The PEO collapse can be explained either by avoiding entropically disfavored chain entanglement or by exceeding the solubility limit of PEO in the space between two micelles (leading to precipitation of PEO on the micelle core). Normally, PEO is expected to show miscibility over a broad (if not even the entire) range of polymer volume fractions in aqueous media. However, depending on block length and due to the presence of the inorganic precursor, a miscibility gap might be formed here for nanomaterials.^108–110^ In fact, the occurrence of the PEO collapse as a microphase separation evokes the existence of a miscibility gap for such nanomaterials, behaving differently than expected from the bulk phase diagram. Indeed, the different coiling of a BCP, especially the PEO block, in dilute solution compared to a corresponding lyotropic phase is a well-established concept in polymer science.^71^

At high polymer volume fractions, the nitrogen physisorption isotherms correspond to an accessible mesopore space, provide a meaningful pore dimension (after postcorrection with a geometrical factor of 1.35) and match the results from SEM (Figure 6B). The presence of isolated voids at low polymer volume fractions also explains why no decent hysteresis loop is observed in mesoporous silica prepared with 57 vol % PEO_441_-*b*-PHA_173_ and PEO_251_-*b*-PHA_093_ (Figure S6), as this concentration marks the borderline of achieving an accessible mesopore network. Furthermore, the porosity *P* (i.e., pore volume) linearly increasing with increasing polymer volume fraction shown in Figure S10G confirms that the entire soft template is incorporated in the inorganic matrix (absence of phase segregation) in each sample and thus confirms that the sample set indeed follows the targeted series of increasing polymer volume fraction.

This study on the soft template amount used for soft templating leads to the conclusion that tailoring the wall size follows two regimes: (1) at low polymer volume fraction, the wall size can be tuned while keeping the pore size constant, whereas (2) at high polymer volume fractions, this simple “raisin-bread model”, in which the micelles (or mesopores) are evenly distributed within a given inorganic volume without being changed (like raisins in a raisin bread), does not hold true anymore. Instead, the wall thickness cannot be tuned independently of the pore size. These two regions partly resolve the initial contradiction between the studies of Stefik and co-workers^47,48,50^ and our studies,^46^ as the Stefik group worked below 60–70 vol % (depending on the oxide’s crystallinity and thus density: 1.9–4.3 g mL^–1^ in the case of niobia)^48,111^ of the PEO-*b*-PHA template (see eq S1 in the Supporting Information for further calculations). In addition, the distinction between persistent and dynamic micelle templating needs to be considered. Since we cannot ensure a trapping of the polymer micelles (ensuring pore size conservation) like in the works of Stefik and co-workers, who applied a dedicated solvent mixture, a glassy micelle core, or cross-linked micelles to guarantee kinetic trapping,^47,48,63,112,113^ a transition from the persistent micelle regime to the dynamic one, allowing chain exchange, might be present here. As a result, a pore size deviation upon varying the template amount could occur due to a transition from persistent-to-dynamic micelles, although the extent (increase by up to 120%) and systematic evolution (no significant fluctuation) of pore size change observed here appears not to be explainable by the presence of dynamic micelles alone when compared with literature values (see Figure S11).^40,47,50,63^ Also, our previous study^46^ revealed that the time period between micelle formation and silica gelation is rather short in this synthesis (30–60 min). While this time scale might be sufficient for micelle equilibration by chain exchange in dilute solution,^61,114^ the rate of chain exchange will be significantly lower during the coassembly of micelles within a concentrating solution in this synthesis, as polymer diffusion and translocation not only depends on the block length^115–117^ but is also decelerated in concentrated solutions,^118,119^ in confinement,^120^ and with increasing viscosity^116,121^ of the solution. During gelation of silica, the viscosity of the mixture increases within minutes by 3 to 4 orders of magnitude, as shown in our previous study,^46,122^ which thus most probably freezes the micelles shortly after their formation. Although an exchange of polymer chains still cannot be excluded and the determination of their rate during soft templating is a matter of future studies, a further mechanism, such as PEO collapse, appears to underlie the pore size increase here.

A quantitative consideration of the wall thickness by SAXS is challenging because overlapping micelles and thus interpenetrating spherical mesopores cause apparent pore-to-pore distances smaller than the mesopore diameter. As a result, the wall thickness determined from the pore-to-pore distance after subtraction of the pore size underestimates the actual wall dimension. Tomography based on STEM, however, is a powerful technique to not only provide a distribution of the wall thickness but also enable in-depth analysis of the pore connectivity. Tomographic reconstruction^123,124^ of projection-angle-dependent STEM images of mesoporous silica prepared with different amounts of PEO_355_-*b*-PHA_171_ within our previous work^46^ yields a three-dimensional model of the pore system. Cutouts of these models are shown in Figure 7A for the samples prepared with 69, 75, and 85 vol %. The models are expected to be accurate 3D representations of the materials due to the high tilt range, high contrast between silica and pores, a low residual alignment error of about 0.5 nm, and optimization of reconstruction and segmentation based on realistic simulations of similar systems.^125,126^ By that, reliable results of nanosized features can be well resolved with a pixel size of 1.26 nm (69 vol %) and 1.62 nm (75 and 85 vol %), respectively. Following a local thickness evaluation (with the corresponding ImageJ plugin),^46,80^ the pore size and wall size can be statistically evaluated by determining the diameter of the largest sphere at any point still fitting in one phase without penetrating the other (see Figure 7E).

**Figure 7:**
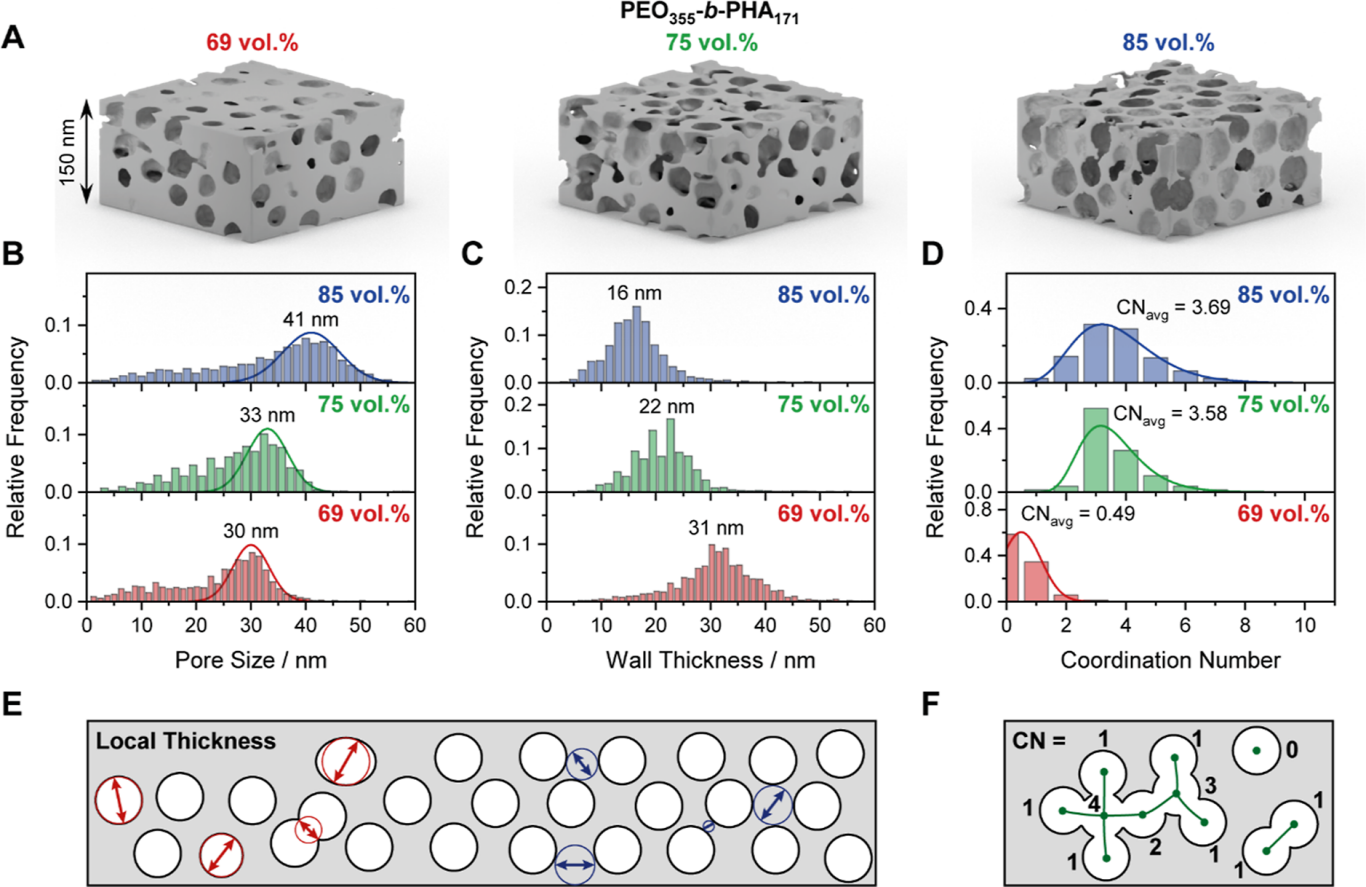
(A) STEM-based 3D reconstructions of mesoporous silica prepared with 69 vol % (left), 75 vol % (middle), and 85 vol % PEO_355_-*b*-PHA_171_ (right) and corresponding distributions of (B) pore size and (C) pore wall thickness from local thickness evaluation as well as (D) number of adjacent mesopores a single pore is connected to through a mesopore window. Mean values of each distribution are given, respectively. The (E) local thickness evaluation as well as the (F) determination of the coordination number are sketched below.

The resulting pore size distributions in Figure 7B confirm the trend observed by SEM for PEO_441_-*b*-PHA_270_-derived mesoporous silica (Figure 6). By increasing the amount of PEO_355_-*b*-PHA_171_ used for soft templating, the mesopore size increases as well. Note that a significant contribution of small mesopores appears in the pore size distribution resulting from pores located at the particle surface and being wrongly assigned to small pore diameters by the algorithm.^46^ Similar to our last study, we therefore used a Gaussian function based on the right part of the distribution (correctly assigned large mesopores) for evaluating the mean pore size (Figure 7B). In addition, the expected decrease in (local) wall thickness can be seen from the distribution in Figure 7C. In all three samples, a homogeneous wall thickness can be found, manifesting as a defined distribution, which steadily shifts to smaller sizes upon increasing the template amount. From 69 to 85 vol %, a thickness decrease by 50% (from 31 to 16 nm) is observed, giving access to a tailoring of wall size over a wide range. For comparison, Sarkar et al. observed a change in wall thickness of around 40% (12 nm vs. 7 nm) by doubling the mass ratio of metal oxide to PEO-*b*-PHA in mesoporous niobium oxide thin films (13 nm mesopores),^47^ which is of a similar order of magnitude.

Beyond the pore and wall size, the physisorption study in Figure 6C hints that the polymer volume fraction has an impact on the pore connectivity and accessibility as well. Electron tomography provides the unique possibility to quantify the connection between the mesopores. Although this technique is restricted to pores above 2 nm due to the pixel size, i.e., connection through micropores cannot be evaluated, a clear difference in connection through large pore windows (>2 nm) can be seen if comparing mesoporous silica templated with low and with high soft template amount (Figure 7D). While at 69 vol %, the majority of mesopores are isolated (coordination number of zero, see Figure 7F) and the remaining 40% of the mesopores are connected to only one or two adjacent mesopores, each mesopore is connected to four neighbors on average in the case of 75 and 85 vol %. This more pronouncedpore connectivity upon increasing the soft template amount is in good agreement with the physisorption study discussed before. Since a fast diffusion within the pore system is governed by not only the number but also especially the the size of connecting pores (necks), an evaluation of the neck size of the mesopores is equally important. Here, the local thickness evaluation of the pore system can be used for determining the bottleneck between two adjacent pores. More precisely, the pore neck size represents the smallest local thickness along the pore skeleton connecting two connected mesopores, as shown by the solid red circles in Figure 8C.^80^ Following this approach, the pore neck size can be evaluated statistically, yielding a neck size distribution (Figure 8A).

**Figure 8:**
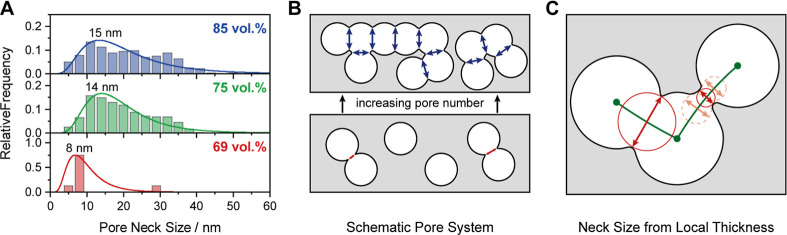
(A) Distribution of the pore neck size of mesoporous silica prepared with 69 vol % (bottom), 75 vol % (middle), and 85 vol % PEO_355_-*b*-PHA_171_ (top). Schematic representation of an exemplary pore system showing (B) the evolution of pore neck size (red and blue) upon increasing pore number, i.e., template amount (bottom to top) and (C) the underlying local thickness evaluation, in which the pore neck size represents the diameter of the smallest sphere along the pore skeleton (green) just touching the silica phase (solid red circle).

Increasing the polymer volume fraction from 69 to 75 vol % not only increases the number of connecting pores but also their diameter. A reliable mean value cannot be given due to the broad distribution (the k-gamma function in Figure 8A acts rather as eye-guidance), and the number of connecting pores at 69 vol % is too small for statistical evaluations, but the few existing pore necks in this sample are still narrower than for silica templated with higher polymer amounts. Consequently, both a quantitative and a qualitative enhancement in pore connectivity can be concluded upon increasing soft template concentration, as sketched in Figure 8B. The comparably large pore neck size, especially in the case of the 75 vol % and 85 vol % samples, evokes an apparent contradiction to the pronounced cavitation phenomenon in physisorption (see isotherms in our previous study^46^ and here in Figure 6 for samples prepared with high template amounts), which suggests a highly restricted pore accessibility. This contradiction can be resolved by the percolation pore model presented in our previous work,^46^ in which a single dead-end of the percolation path composed of well-connected pores renders all pores of this path poorly accessible for the adsorptive. Taking the 11 mesopores of the scheme in Figure 7F as an example, the spherical pores interconnect to three subnetworks (i.e., percolation paths shown as green lines). Within these percolation paths, the pores are well-connected by the large pore necks observed in Figure 8A, but pore connectivity among individual paths and with the exterior of the particle is followed by small PEO-single-chain-induced micropore channels of 1–2 nm in diameter, restricting evaporation from the pores and causing the pronounced cavitation in the adsorption isotherms.^46^ Thus, the pore neck size evaluation here is in good alignment with the previously proposed pore model.

### Extending the Mesopore Size Regime

The concentration study with PEO_441_-*b*-PHA_270_ and PEO_355_-*b*-PHA_171_ as soft templates showed that the amount of BCP in soft templating influences the pore size, wall thickness, and pore connectivity. Although the pore size increase at large polymer volume fractions renders an independent wall size tuning in this regime impossible, the influence on the pore size offers the opportunity to reach even larger spherical pores than those concluded from Figure 5. Therefore, the entire polymer library was used once more for templating silica, but this time employing 85 vol % of each soft template. As shown in the SEM images in Figure S12, a similar pore morphology is obtained as in the case of 57 vol % template (Figure 4): ordered spherical pores are achieved with large PEO block lengths (*n* = 441 and 251) only, while block copolymers with short PEO and long PHA block led to rather macro- or nonporous silica. The template PEO_047_-*b*-PHA_096_ represents a special case again featuring some small mesopores (12 nm in size) in SEM but overall a low porosity according to nitrogen physisorption (Figure S13), featuring a mode mesopore diameter of around 27 nm. As discussed above, this might be due to the fact that this BCP is at the border of forming stable micelles. Regarding the remaining templates yielding spherical pores, a high, well-defined porosity is obtained, as confirmed by the nitrogen physisorption isotherms in Figure S13. However, in contrast to the samples prepared with 57 vol %, the pore size is twice as high, demonstrating the pore size increase to be a general trend of PEO-*b*-PHA soft templates. As explained above and shown in Table S5, the pore diameters determined from SEM, physisorption, and SAXS are in good alignment for pore sizes below 35 nm, but for larger pores, physisorption and SAXS become inaccurate. Hence, the SEM-based pore diameter was used for quantitative interpretations. Again, a plot of the pore size *d*_p_ against the PHA block length *m* reveals clearly a linear increase with increasing block length (Figure 9A) following the relation in eq 4

4 $$d_{p}\left(85\%\right)=0.14\;nm\cdot m+17.3\;nm$$

**Figure 9:**
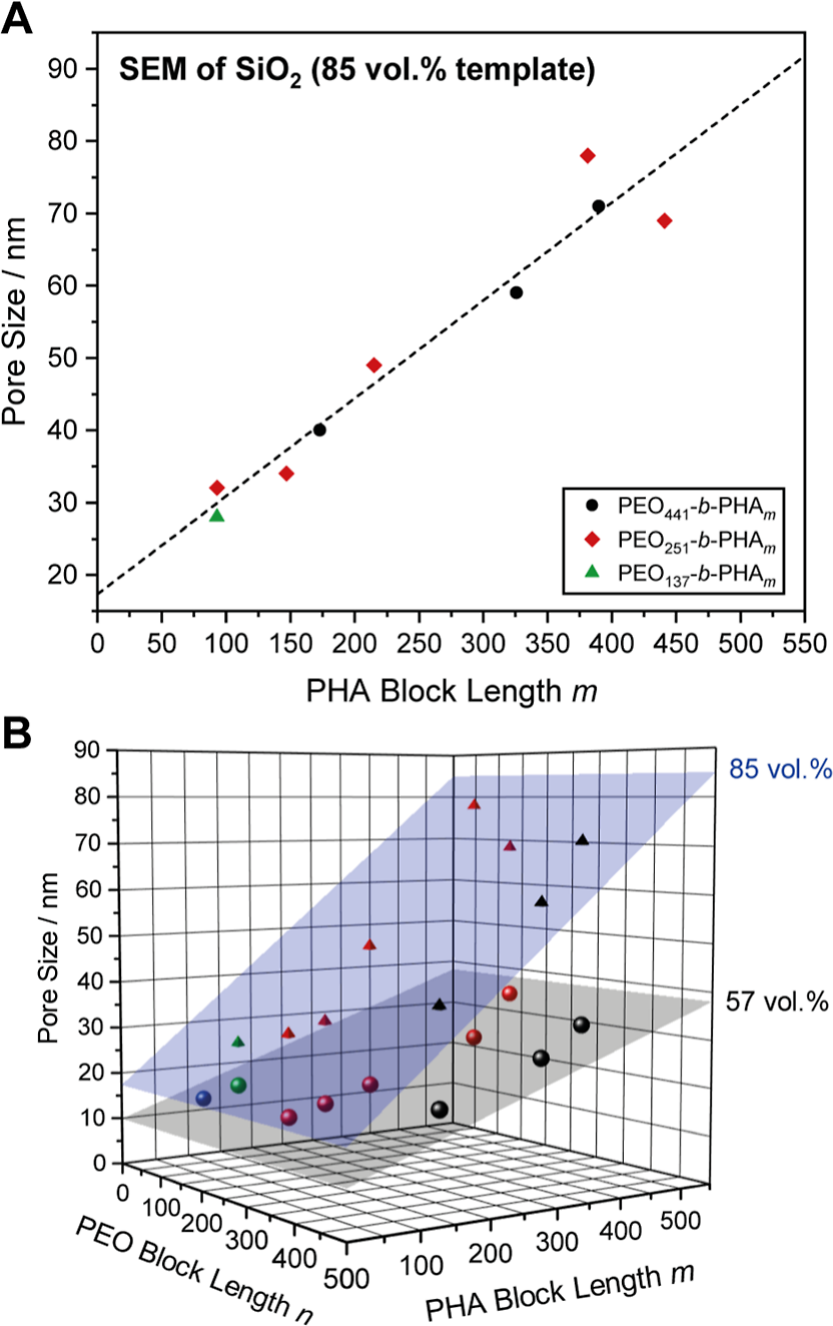
(A) SEM-based pore size of mesoporous silica prepared using 85 vol % PEO_*n*_-*b*-PHA_*m*_ with a PEO block length of *n* = 441 (black), 251 (red), and 137 (green), plotted against the PHA block length from NMR. (B) 3D Plot of the mesopore size from SEM against PEO and PHA block length for samples prepared with 57 (spheres) and 85 vol % (pyramids) soft template, including a respective fit (gray and blue plane) spanning the accessible pore size regime.

Indeed, both offset and slope of the linear regression are twice as large as those in the case of the 57 vol % soft template, implying a PEO contribution of 17 nm to the mesopore size and a pore size increase of 14 nm per 100 HA units added to the PHA block of the soft template. The larger PEO contribution supports the hypothesis of a PEO collapse upon increasing polymer concentration suggested before (Figure 6) and will be discussed in more detail in the next section.

Considering that the pore size obtained with 57 vol % polymer can be regarded as the “natural” pore size achievable with the PEO-*b*-PHA copolymers according to the constant regime (1) in Figure 6A, the values resulting from 85 vol % (“expanded” pore size) represent the maximum pore size a template can introduce because higher quantities of soft template cannot be dissolved in the precursor solution. Thus, by adjusting the template concentration, a pore size tuning from 15 to 80 nm is possible with the PEO-*b*-PHA template family in the case of silica prepared in ethanolic solution. This regime represents the volume spanned by the boundary regression planes in the 3D plot shown in Figure 9B. Based on our last study with mesoporous zirconium oxide possessing the same mesopore size as silica,^46^ these results might be generalized, stating that PEO-*b*-PHA can cover a mesopore size range from 10 to 80 nm, although soft template amounts have to be given in units of vol % in order to compare it along different pore skeleton materials.

To ensure that no polymer residues in the mesoporous silica samples hamper porosity evaluations, a typical (noncalcined) template/silica hybrid gel (57 vol % PEO_441_-*b*-PHA_326_) was exposed to the heating procedure used to prepare all samples and studied by thermogravimetric analysis (Figure S16B). Following the evolution of the mass signals assigned to released water and carbon dioxide, decomposition of the soft template starts at 250 °C and is completed at the beginning of the 500 °C dwell step. The weight loss of 36 wt % during the transformation of the hybrid material to the final oxide matches well the expected theoretical loss of 39 wt % (23.0 mg of polymer vs. 35.2 mg of SiO_2,_ assuming full conversion of TEOS). To back up a full removal of the soft template, an already calcined mesoporous sample prepared with high template amounts (85 vol %) as an extreme case was heated to 1000 °C and gravimetrically studied (Figure S16A). Since only a mass loss due to the release of adsorbed water at 110 °C, but no significant intensity of the CO_2_ mass signal can be observed, a complete PEO-*b*-PHA decomposition can be concluded.

### Contribution of PEO to Micro- and Mesoporosity

Based on these insights, we are able to explain and predict the influence of the PHA and PEO block lengths as well as the polymer concentration on the porosity. In the following, we address the potential underlying reason suggested to be a PEO collapse , i.e. the formation of a layer of poorly solvated PEO chains, on the micelle core. As proposed before, the micelle core, being composed of the PHA block and collapsed PEO on top, is expected to build the mesopore, while residual PEO in the micelle corona might penetrate the inorganic wall, forming micropores. Therefore, the contribution of the micropore and mesopore volume to the total pore volume appears as a suitable descriptor to confirm a possible PEO collapse. In detail, the mesopore fraction Φ_meso_ is used for quantification here and describes the share of the mesopore volume *V*_p,meso_ from physisorption with respect to the total pore volume (sum of mesopore volume and micropore volume *V*_p,micro_), as described in eq 5

5 $$\Phi_{meso}=\frac{V_{p,meso}}{V_{p,meso}+V_{p,micro}}$$

In the case of ordered mesoporous silica, the cumulative pore size distribution from physisorption typically possesses a distinct shape, as shown in the inset (top right) of Figure 10A. So, *V*_p,micro_, and *V*_p,meso_ can be extracted from the respective step heights. These volumes can be inserted into eq 5, yielding the relative share Φ_meso_. Applying this procedure to the pore size distributions shown in Figures S7 and S14, respectively, and plotting the obtained values against the respective PHA block length leads to Figure 10. Within this templating study, three parameters were changed: (1) the PHA block length, (2) the PEO block length, and (3) the BCP concentration. Each of them influences the mesopore fraction Φ_meso_, as summarized in the inset of Figure 10B.

1 An increase in PHA block length while keeping the PEO block length constant is expected to swell the micelle core. Thus, the mesopore volume increases, whereas the micropore volume remains unchanged, leading to an increase in Φ_meso_ with increasing block length *m*, which is indeed observed for both concentrations and for all PEO block lengths. This effect is more pronounced in the case of the 57 vol % soft template (overall increase of ∼20% versus <10% in the case of 85 vol % BCP), which can be attributed to a secondary effect being superimposed: Upon increasing the PHA block length, the micelle core expands, and by that, more PHA is exposed to the surface of the micelle core (increase in micelle core surface area). In order to ensure screening of the hydrophobic core from the hydrophilic environment, more PEO collapses on the core, and less PEO will contribute to the micropore volume, resulting in a further increase of Φ_meso_. In the case of 85 vol %, a high amount of PEO is already collapsed on the micelle core, and thus additional collapse upon increasing *m* does not appear. As a result, this secondary effect is less pronounced or no longer occurs.
2 Increasing the PEO block length, keeping the PHA block length constant, should enlarge the micropore volume, leaving the mesopore volume untouched. Consequently, the mesopore fraction Φ_meso_ should be smaller for samples prepared with a larger PEO block (unless there is no phase separation because PEO cannot be solvated fully by the solvent/precursor). Looking at Figure 10A, this trend is less apparent due to the data points being more scattered than for 85 vol %, but regarding the latter in Figure 10B this effect is slightly recognizable. Although the difference is rather small, Φ_meso_ increases along the sequence of PEO_*n*_-*b*-PHA_*m*_-derived silica of decreasing PEO block length (from *n* = 441 to 251 to 137).
3 The last effect revolves around the variation of the template concentration and, by that, tackles the central hypothesis of PEO collapse. A collapse of EO units on the core is expected to cause an increase in mesopore volume (pore size increases) parallel to a decrease in micropore volume (fewer EO units contribute to single-chain templating). As a result, the mesopore contribution Φ_meso_ should increase. Comparing Figure 10A with Figure 10B, this effect appears to be particularly striking: all samples templated with an 85 vol % soft template show a larger Φ_meso_ than all silica samples prepared with a 57 vol % polymer. On average, Φ_meso_ increases by 20 percentage points upon increasing the polymer volume fraction from 57 to 85 vol %. Similarly, a continuous increase in Φ_meso_ simultaneous to a starting pore size increase above 60 vol % is visible in PEO_441_-*b*-PHA_270_-derived silica in the systematic concentration row (Figure S10G). These observations confirm that Φ_meso_ is a suitable descriptor for investigating the templating behavior and strongly support the hypothesis of PEO collapse causing the pore size increase at high polymer volume fractions.

**Figure 10:**
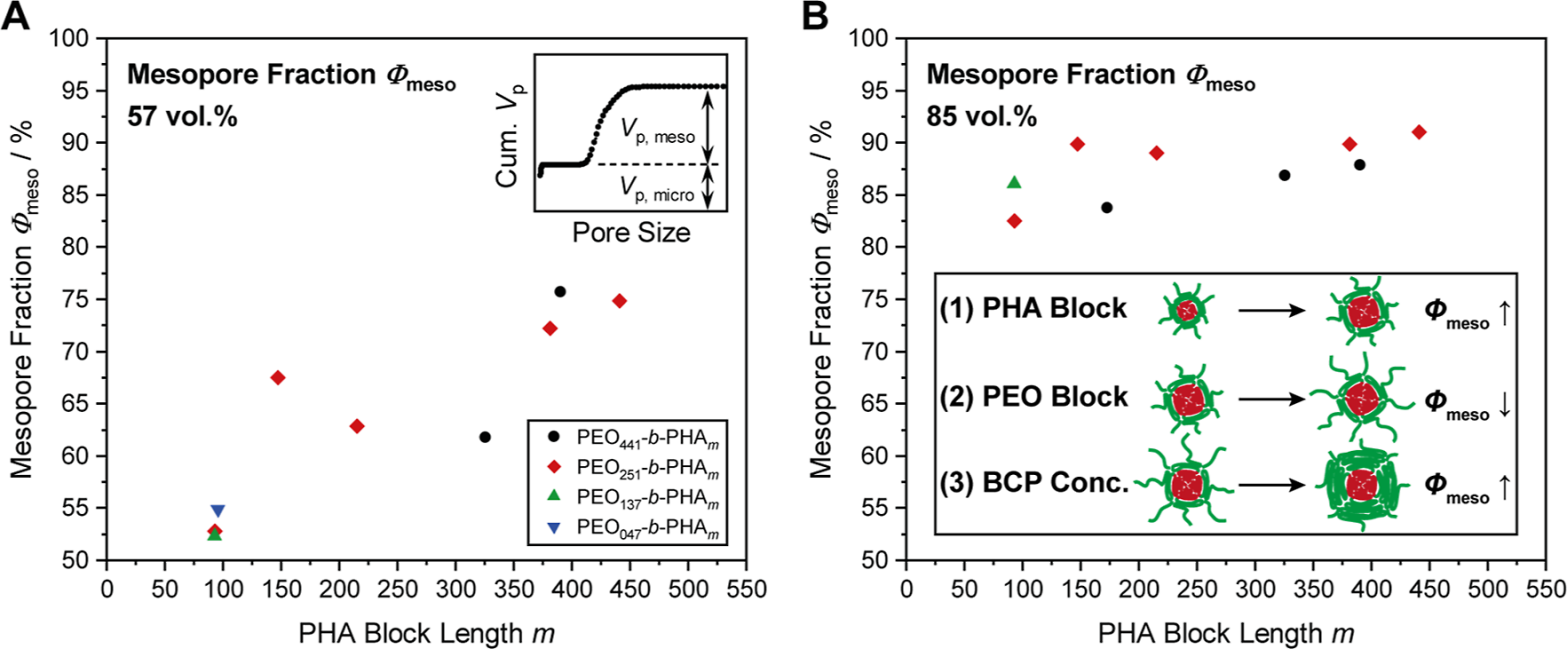
Mesopore fraction as a ratio of mesopore volume to total pore volume (that means the ratio of *V*_p,meso_ to the sum of *V*_p,meso_ and *V*_p,micro_ shown in the inset) of mesoporous silica prepared with (A) 57 vol % and (B) 85 vol % PEO_*n*_-*b*-PHA_*m*_ with a PEO block length of *n* = 441 (black), 251 (red), 137 (green), and 47 (blue) plotted against the PHA block length from NMR. Schematic illustration of the expected trends of the mesopore fraction with (1) increasing PHA block length, (2) increasing PEO block length, and (3) increasing BCP concentration is shown in the inset on the right.

The hypothesis of a constant amount of collapsed PEO in order to shield the PHA core from the hydrophilic surroundings is supported by the constant offsets in Figures 5 and 9 for all PEO block lengths and the slight PEO block influence on Φ_meso_ (trend (2) in Figure 10B). However, if this hypothesis holds true and the entire residual PEO block penetrates the inorganic pore wall, the polymer volume fraction at which the pore size starts to increase should depend on the PEO block length. As illustrated in Figure 11A, a higher soft template concentration is required to initiate a micelle–micelle interaction in the case of a shorter PEO block than in the case of a longer one. While the latter faces PEO chain entanglement at a lower polymer concentration, a BCP with a shorter PEO block still enables a wall size tuning under preservation of the pore size.

**Figure 11:**
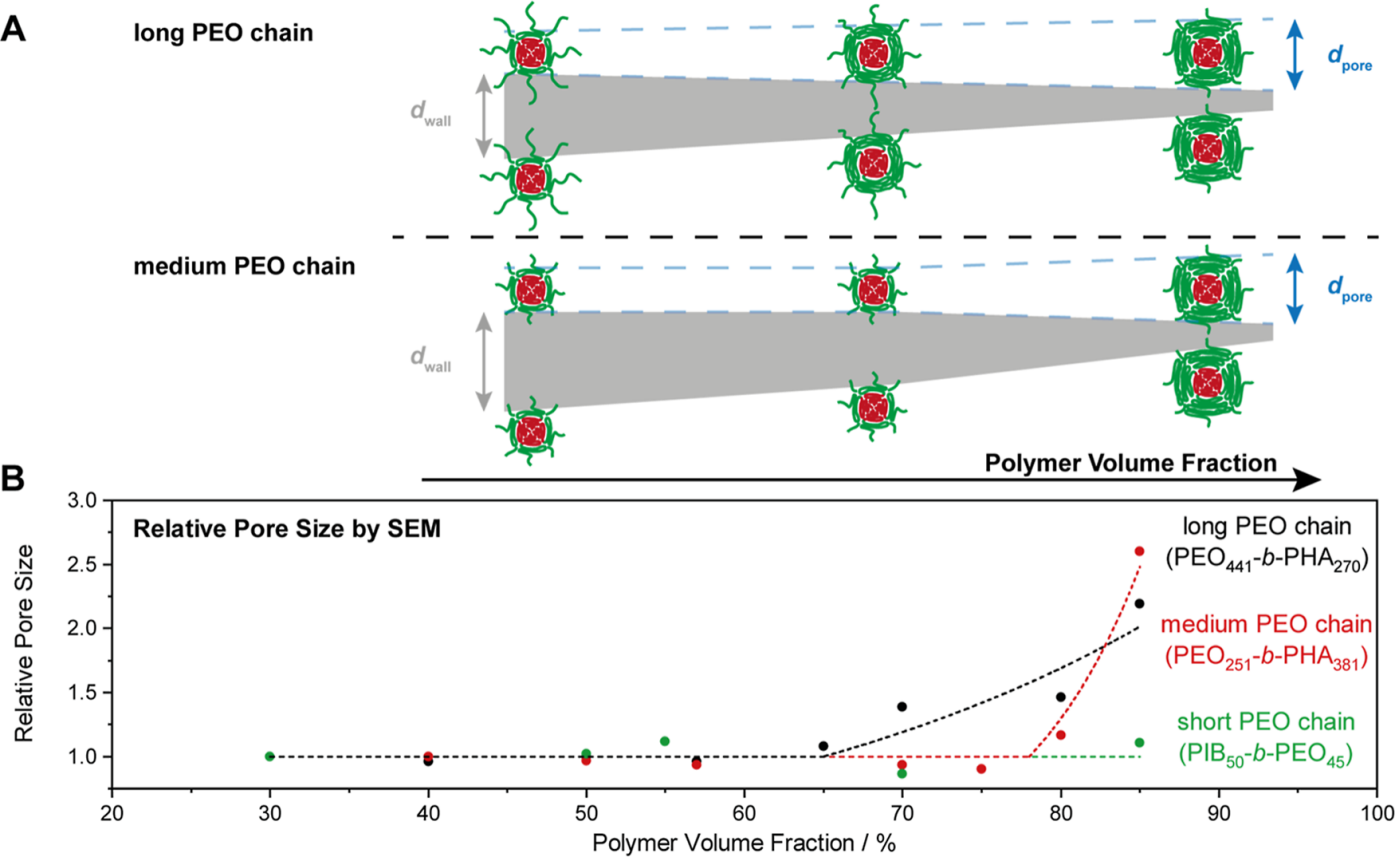
(A) Schematic evolution of pore and wall size with increasing polymer concentration, i.e., decreasing micelle-to-micelle distance for two different PEO block lengths. (B) SEM-based relative pore size (pore size divided by the initial pore size at 30 vol %) versus the polymer concentration of PEO_441_-*b*-PHA_270_ (black), PEO_251_-*b*-PHA_381_ (red), and PIB_50_-*b*-PEO_45_ (green) used for soft templating of mesoporous silica.

To investigate this postulation, the former concentration study with PEO_441_-*b*-PHA_270_ (Figure 6) is extended by a similar series with a BCP possessing a shorter PEO block (PEO_251_-*b*-PHA_381_) and a template with a very short PEO block (PIB_50_-*b*-PEO_45_); the latter is preferred over PEO-*b*-PHA templates with a PEO block length of 137 and 47, as they do not lead to a well-ordered pattern of spherical mesopores over a broad concentration range.

Since the three soft templates of this concentration study induce mesopores of different pore size regimes, a comparison is only possible after normalization of the average pore size by the initial one observed at the lowest polymer volume fraction. By evaluation of the SEM images (Figure S17) of the mesoporous silica samples templated with PEO_251_-*b*-PHA_381_ and poly­(isobutylene)-*block*-poly­(ethylene oxide) (PIB_50_-*b*-PEO_45_) and respective normalization, the evolution of the relative pore size along increasing polymer concentration can be compared with the former concentration study. As shown in Figure 11B, the transition from regime (1) of constant pore size to the pore size increase in regime (2) depends on the PEO block length. While the soft template with long PEO block starts to show a BCP-concentration-induced pore size increase from 65 vol % onward, the medium PEO block shifts this behavior to higher polymer volume fractions (∼75 vol %), and with a short PEO block, no influence of the concentration on the pore size is observed at all. This study not only confirms the aforementioned hypothesis of a constant PEO collapse (already suggested from the constant offset in Figures 5 and 9) but also implies that the regime of wall size tuning under pore size conservation can be adjusted by the choice of the PEO block length. This is an important feature for studies in which the influence of only the wall size needs to be investigated. In conclusion, a large PEO block is important to guarantee solubility of the soft template for a large pore size but limits the regime of an independent wall size tuning.

## Conclusion

In this study, we systematically varied the PEO and PHA block lengths and concentration of poly­(ethylene oxide)-*block*-poly­(hexyl acrylate) (PEO-*b*-PHA) block copolymers in soft templating of silica. We found this soft template class to produce spherical mesopores in a pore diameter range of 10–80 nm with a change by 6 nm per 100 HA units at low and 14 nm per 100 HA units at high template concentrations. Applying a polymer library of 17 copolymers with four different PEO and tailored PHA block lengths shows a large PEO block to be necessary to avoid vesicle formation and to ensure ordered mesoporous morphologies. In the latter, the pore wall thickness scales inversely with the soft template concentration according to SEM, nitrogen physisorption, SAXS, and electron tomography. In addition, the higher the template concentration, the more enhanceder the pore connectivity and the larger the mesopores exceeding a certain threshold (of about 60 vol % in the case of the largest PEO block). This threshold marks the soft template concentration, below which the wall size can be tailored under preservation of the pore size (Figure 12). Using three different block copolymers featuring different PEO block lengths as soft templates, we demonstrate the threshold concentration shifts to higher values when the PEO block is shorter, which enlarges the concentration range of independent wall size tuning. A pore volume analysis confirmed that a partial collapse of the PEO block in the micelle corona onto the PHA core causes the pore size to increase above the threshold template concentration. This PEO collapse leading to a loss of microporosity represents an important effect governing the pore size, which needs to be considered in soft templating with amphiphilic block copolymers.

**Figure 12:**
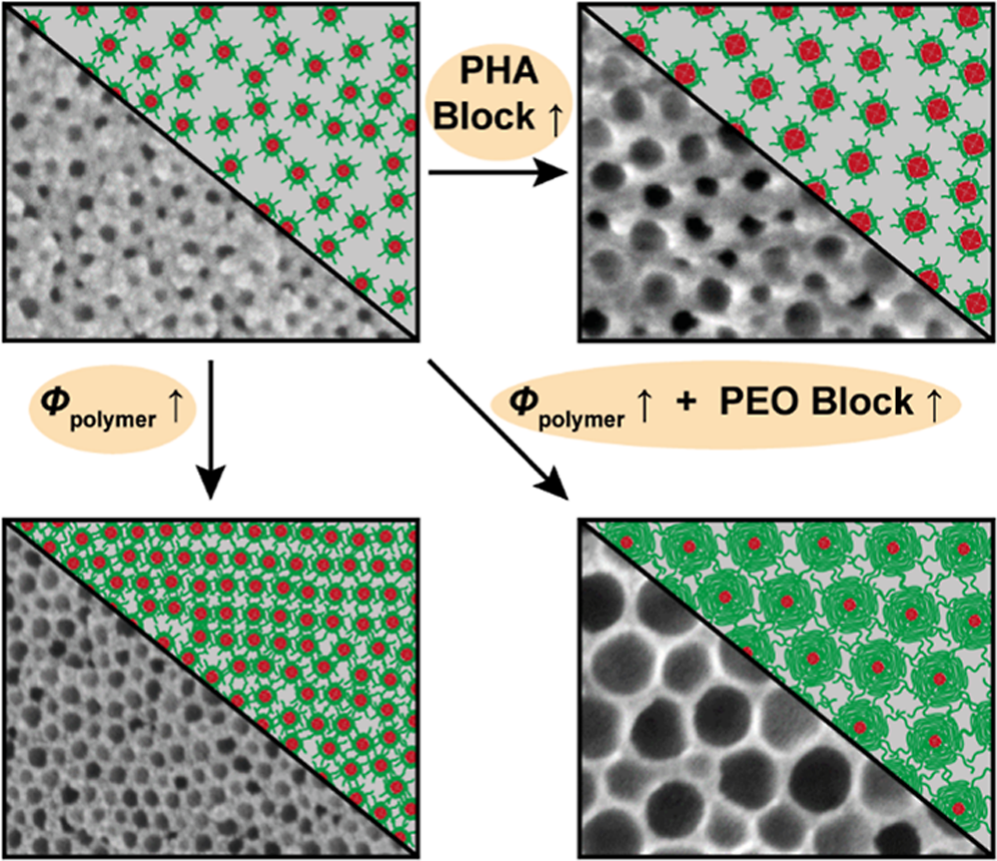
Schematic summary of the observed trends in soft templating of silica with PEO-*b*-PHA templates, in which the sketched micelle arrangement is contrasted to exemplary SEM images after calcination. An increase in PHA block size (top left to top right) increases the mesopore size, while an increase in template concentration yields a decrease in pore wall thickness under conservation of pore size in the case of small PEO blocks (top left to bottom left). If the PEO block is sufficiently large, an increasing template concentration additionally leads to an increase in pore size due to the collapse of the PEO block on the PHA core (top left to bottom right).

Upon varying the soft template amount, we identified a pore volume fraction of 55–60 vol % according to physisorption (considering micro- and mesopores) and 75 vol % according to tomography (considering only mesopores) to be necessary to obtain an accessible and interconnected pore system. Compared with a cubic close packing possessing a filled volume of 74%, the threshold determined by tomography considering only the spherical mesopores (and no micropores) becomes geometrically reasonable. This pore fraction can be regarded as the percolation threshold, above which enough pores are interconnected to form a continuously open pore network and might be a universal guide for mesoporous materials with spherical pores in general, in which an accessible pore system is (un)­desired.

The quantitative pore size relations and the interplay between soft template concentration and wall thickness, pore size, and pore connectivity studied here represent important experimental guidelines to achieve a targeted porosity deliberately and to set up systematic studies on porosity–property relationships.

## Supplementary Material





## Notes

Image stacks of the STEM-based reconstructions of the silica particles (ZIP)
Additional experimental details on the reagent amounts; ^1^H NMR spectra, GPC traces, and DLS spectra of all block copolymers; SEM images, nitrogen isotherms (including pore size distributions), and SAXS curves of all mesoporous silica powders; polymer characterization and pore size evaluation of all samples in tabular form (PDF)
