# Supplementary material for: Porosity Tuning in Soft-templated Mesoporous Silica: The Influence of Block Copolymer Composition and Concentration
Source: Langmuir. 2025 Nov 13;41(46):30948–67. doi: 10.1021/acs.langmuir.5c02750 (PMC12659430; doi:10.1021/acs.langmuir.5c02750)
Supplement: Supplementary file 2 [file la5c02750_si_002.pdf]

Supporting Information

Porosity Tuning in Soft-templated Mesoporous Silica: The Influence of Block Copolymer Composition and Concentration

*Lysander Q. Wagner[a,b], Frederik Breckwoldt[a,b], Xiaohui Huang[c,d], Christian Kübel[c,d,e], Xiaoyin Cheng[f], Katja Schladitz[f], and Bernd M. Smarsly[a,b]**

a. Institute of Physical Chemistry, Justus Liebig University, Heinrich-Buff-Ring 17, D-35392 Giessen, Germany

b. Center of Materials Research, Justus Liebig University, Heinrich-Buff-Ring 16, D-35392 Giessen, Germany

c. Institute of Nanotechnology, Karlsruhe Institute of Technology, Hermann-von-Helmholtz-Platz 1, D-76344 Eggenstein-Leopoldshafen, Germany

d. Department of Materials and Earth Science, Technical University Darmstadt, Peter-Grünberg-Straße 2, D-64287 Darmstadt, Germany

e. Karlsruhe Nano Micro Facility, Karlsruhe Institute of Technology, Hermann-von-Helmholtz-Platz 1, D-76344 Eggenstein-Leopoldshafen, German

f. Fraunhofer Institute for Industrial Mathematics, Fraunhofer-Platz 1, D-67663 Kaiserslautern, Germany

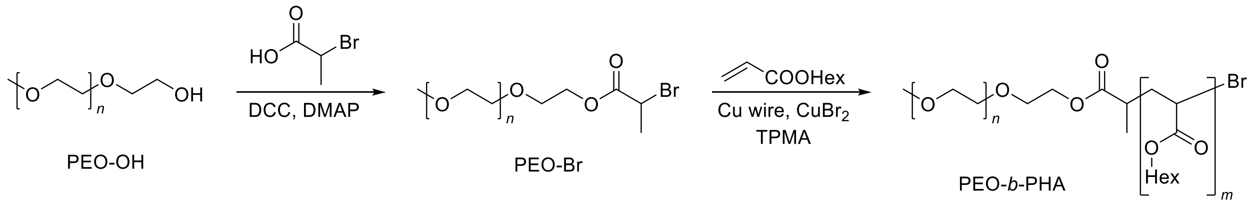

Scheme S1. Two-step synthesis of PEO-*b*-PHA block copolymers by a SARA ATRP.

Table S1. Overview on the amounts of macroinitiator (PEO-Br), monomer (hexyl acrylate), ligand (TPMA), $CuBr_2$, and solvent (DMF) used for the SARA ATRP reactions in this study.

Sample	PEO-Br	Monomer	TPMA	$CuBr_2$	DMF
**PEO$_{441}$-*b*-PHA$_{390}$**	2001 mg	8.30 mL	88 mg	35 mg	6 mL
**PEO$_{441}$-*b*-PHA$_{326}$**	2000 mg	6.60 mL	89 mg	34 mg	6 mL
**PEO$_{441}$-*b*-PHA$_{270}$**	1999 mg	5.60 mL	89 mg	35 mg	6 mL
**PEO$_{441}$-*b*-PHA$_{173}$**	1999 mg	3.51 mL	88 mg	34 mg	6 mL
**PEO$_{251}$-*b*-PHA$_{441}$**	500 mg	4.35 mL	44 mg	17 mg	5 mL
**PEO$_{251}$-*b*-PHA$_{381}$**	1000 mg	6.71 mL	88 mg	33 mg	6 mL
**PEO$_{251}$-*b*-PHA$_{215}$**	500 mg	2.41 mL	43 mg	17 mg	5 mL
**PEO$_{251}$-*b*-PHA$_{147}$**	500 mg	1.83 mL	44 mg	17 mg	5 mL
**PEO$_{251}$-*b*-PHA$_{093}$**	2001 mg	3.37 mL	175 mg	67 mg	10 mL
**PEO$_{137}$-*b*-PHA$_{544}$**	300 mg	3.35 mL	9 mg	3 mg	5 mL
**PEO$_{137}$-*b*-PHA$_{517}$**	300 mg	3.35 mL	17 mg	7 mg	5 mL
**PEO$_{137}$-*b*-PHA$_{451}$**	500 mg	5.58 mL	7 mg	3 mg	5 mL
**PEO$_{137}$-*b*-PHA$_{381}$**	500 mg	5.60 mL	87 mg	33 mg	5 mL
**PEO$_{137}$-*b*-PHA$_{283}$**	500 mg	4.92 mL	87 mg	34 mg	5 mL
**PEO$_{137}$-*b*-PHA$_{227}$**	501 mg	3.67 mL	88 mg	35 mg	5 mL
**PEO$_{137}$-*b*-PHA$_{093}$**	500 mg	1.73 mL	87 mg	34 mg	5 mL
**PEO$_{047}$-*b*-PHA$_{468}$**	250 mg	9.67 mL	36 mg	14 mg	6 mL
**PEO$_{047}$-*b*-PHA$_{096}$**	1000 mg	7.80 mL	160 mg	63 mg	5 mL

Table S2. Amount of each soft template used for preparing mesoporous silica of varying porosity (polymer volume fraction Φ in vol.%).

Soft Template	57 vol.%	85 vol.%	Soft Template	Amount	Φ
**PEO$_{441}$-*b*-PHA$_{390}$**	23 mg	98 mg	**PEO$_{441}$-*b*-PHA$_{270}$**	7 mg	30 vol.%
**PEO$_{441}$-*b*-PHA$_{326}$**	23 mg	98 mg		11 mg	40 vol.%
**PEO$_{441}$-*b*-PHA$_{270}$**	23 mg	98 mg		17 mg	50 vol.%
**PEO$_{441}$-*b*-PHA$_{173}$**	23 mg	99 mg		32 mg	65 vol.%
**PEO$_{251}$-*b*-PHA$_{441}$**	23 mg	97 mg		41 mg	70 vol.%
**PEO$_{251}$-*b*-PHA$_{381}$**	23 mg	98 mg		69 mg	80 vol.%
**PEO$_{251}$-*b*-PHA$_{215}$**	23 mg	98 mg	**PEO$_{251}$-*b*-PHA$_{381}$**	12 mg	40 vol.%
**PEO$_{251}$-*b*-PHA$_{147}$**	23 mg	99 mg		17 mg	50 vol.%
**PEO$_{251}$-*b*-PHA$_{093}$**	24 mg	100 mg		40 mg	70 vol.%
**PEO$_{137}$-*b*-PHA$_{544}$**	23 mg	97 mg		52 mg	75 vol.%
**PEO$_{137}$-*b*-PHA$_{517}$**	23 mg	97 mg		69 mg	80 vol.%
**PEO$_{137}$-*b*-PHA$_{451}$**	23 mg	97 mg	**PIB$_{50}$-*b*-PEO$_{45}$**	7 mg	30 vol.%
**PEO$_{137}$-*b*-PHA$_{381}$**	23 mg	98 mg		16 mg	50 vol.%
**PEO$_{137}$-*b*-PHA$_{283}$**	23 mg	98 mg		20 mg	55 vol.%
**PEO$_{137}$-*b*-PHA$_{227}$**	23 mg	97 mg		38 mg	70 vol.%
**PEO$_{137}$-*b*-PHA$_{093}$**	23 mg	99 mg		91 mg	85 vol.%
**PEO$_{047}$-*b*-PHA$_{468}$**	23 mg	97 mg			
**PEO$_{047}$-*b*-PHA$_{096}$**	23 mg	99 mg			

Table S3. Experimental details of the STEM-based tomography.

Sample	Tilt Range	Pixel Size	Residual Error	Voxel Size	Ball Radius
**69 vol.%**	–74° to 66°	0.63 nm	0.477 nm	$(1.26\ \text{nm})^3$	40 px
**75 vol.%**	–74° to 68°	0.81 nm	0.573 nm	$(1.62\ \text{nm})^3$	30 px

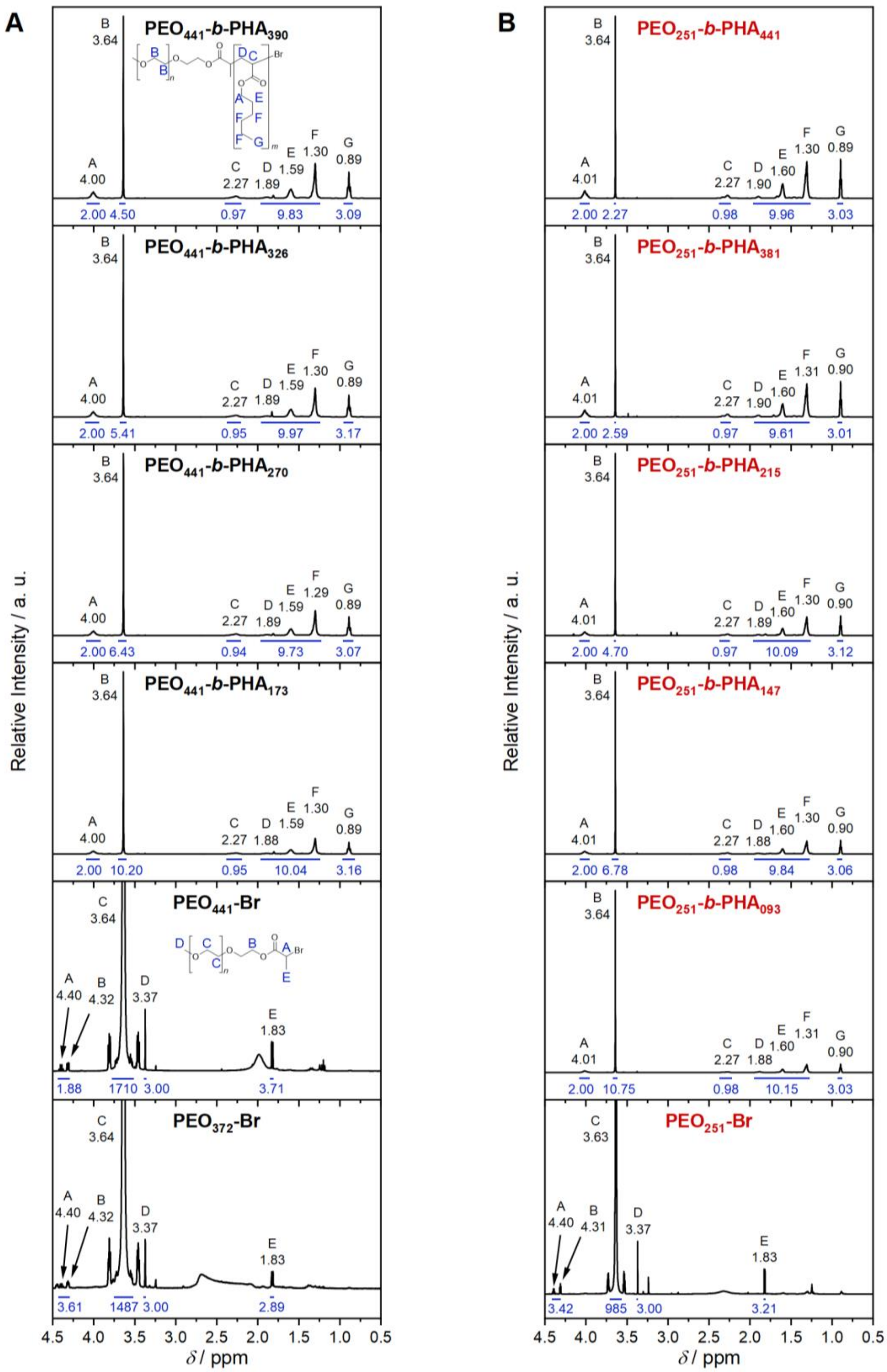

Figure S1. ^{1}H NMR (400 and 700 MHz) spectra of PEO-Br macroinitiators (20 kDa, black and 10 kDa, red) and PEO-*b*-PHA block copolymers based on them in $CDCl_3$.

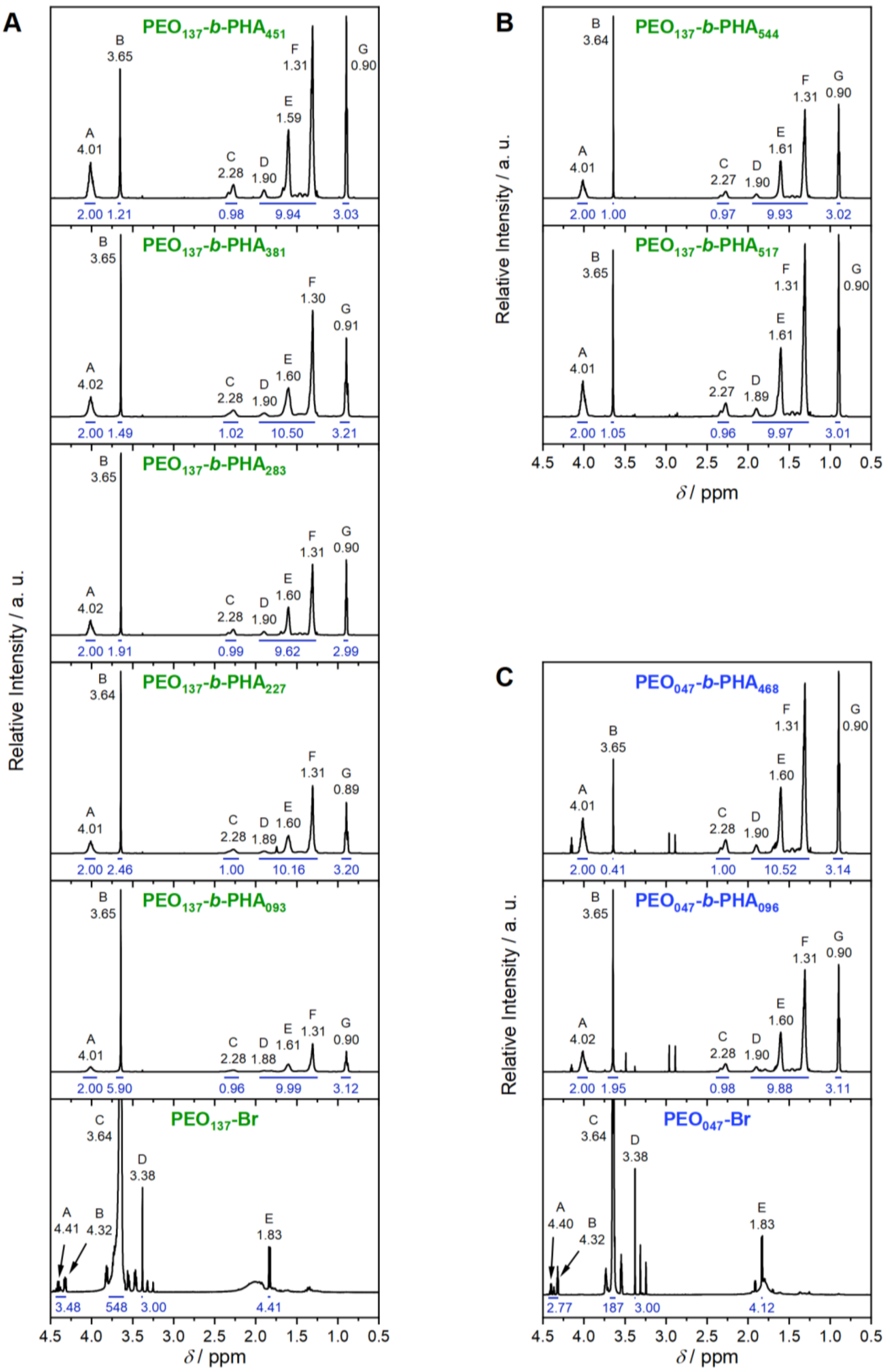

Figure S2. ^{1}H NMR (400 and 700 MHz) spectra of PEO-Br macroinitiators (5 kDa, green and 2 kDa, blue) and PEO-*b*-PHA block copolymers based on them in $CDCl_3$.

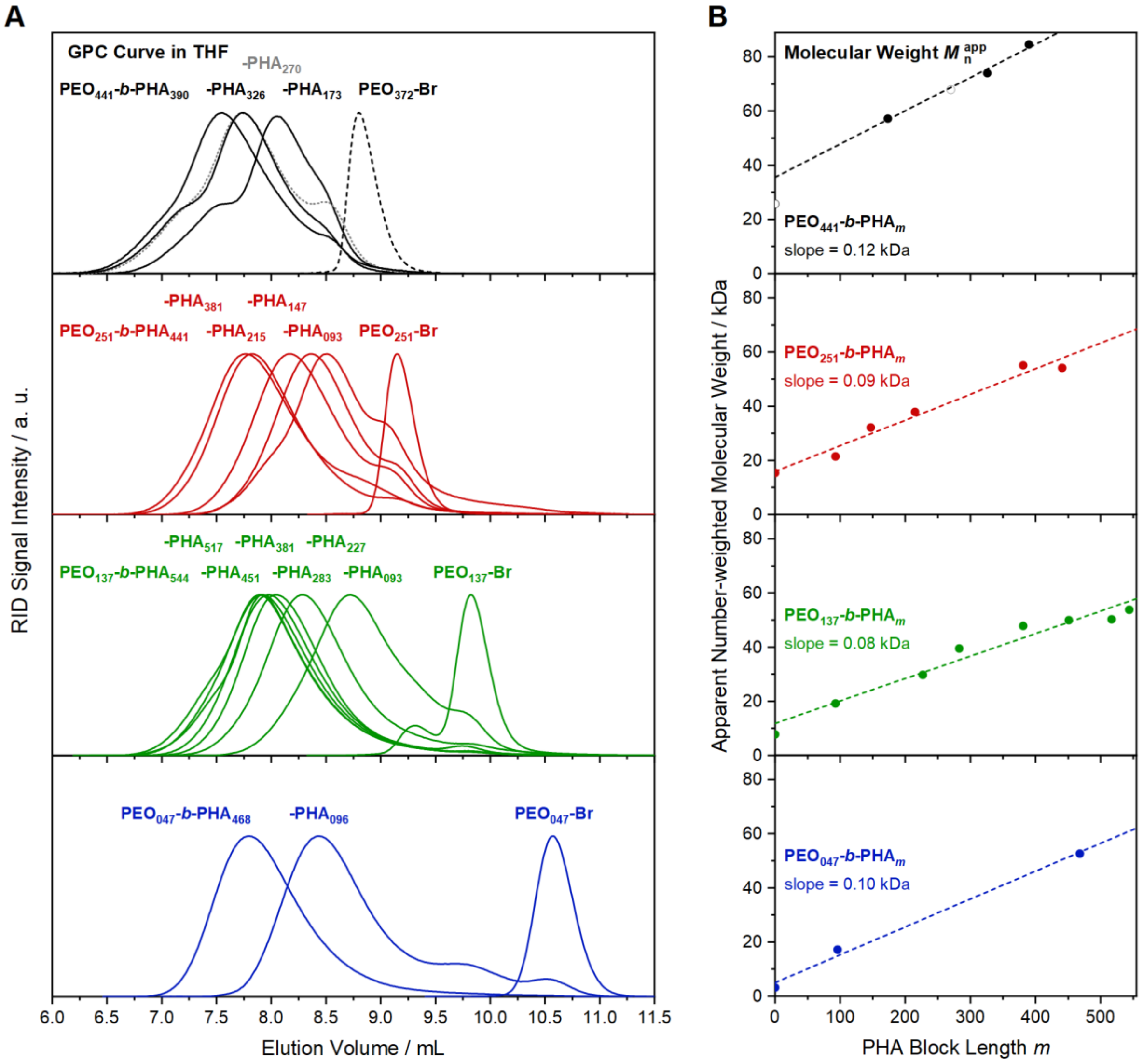

Figure S3. (A) GPC RI traces of the PEO-*b*-PHA block copolymers and the underlying macroinitiators of varying PEO block length (441 EO units in black, 251 in red, 137 in green, and 47 in blue) in THF. Labelling follows increasing PHA block length from right to left in accordance with the shift of the corresponding curve to lower elution volumes. The GPC curve of PEO_{372}-Br (dashed line) is given as references due to the absence of sufficient amounts of PEO_{441}-Br for characterization. (B) Plot of each apparent number-weighted molecular weight from GPC against the NMR-based PHA block length with a linear fit (dashed line) each. Open symbols indicate the reference PEO_{372}-Br sample and the block copolymer excluded from the soft templating matrix.

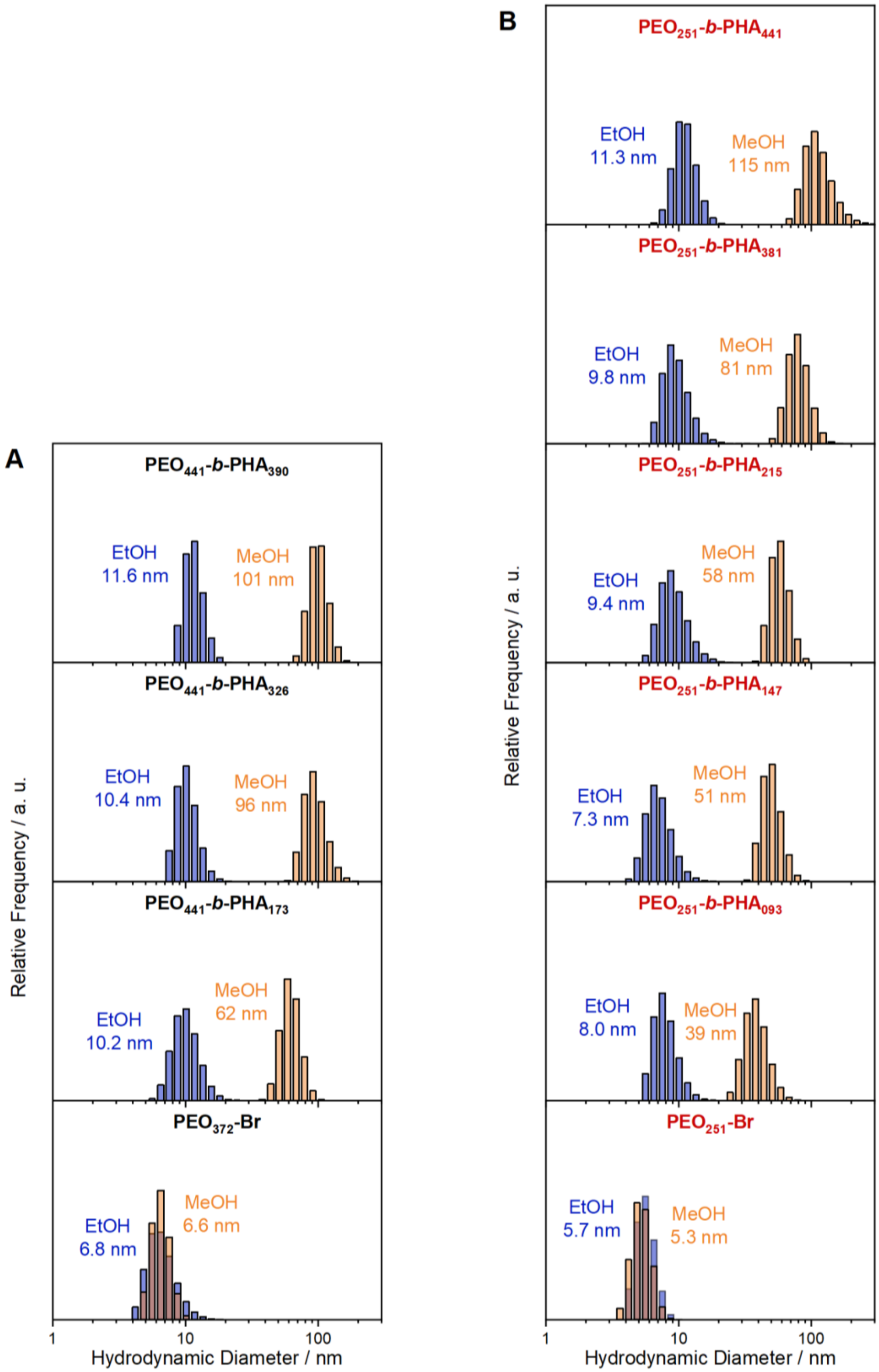

Figure S4. DLS data of PEO-Br macroinitiators (20 kDa and 10 kDa) and PEO-*b*-PHA block copolymers based on them in ethanol (blue) and in methanol (orange).

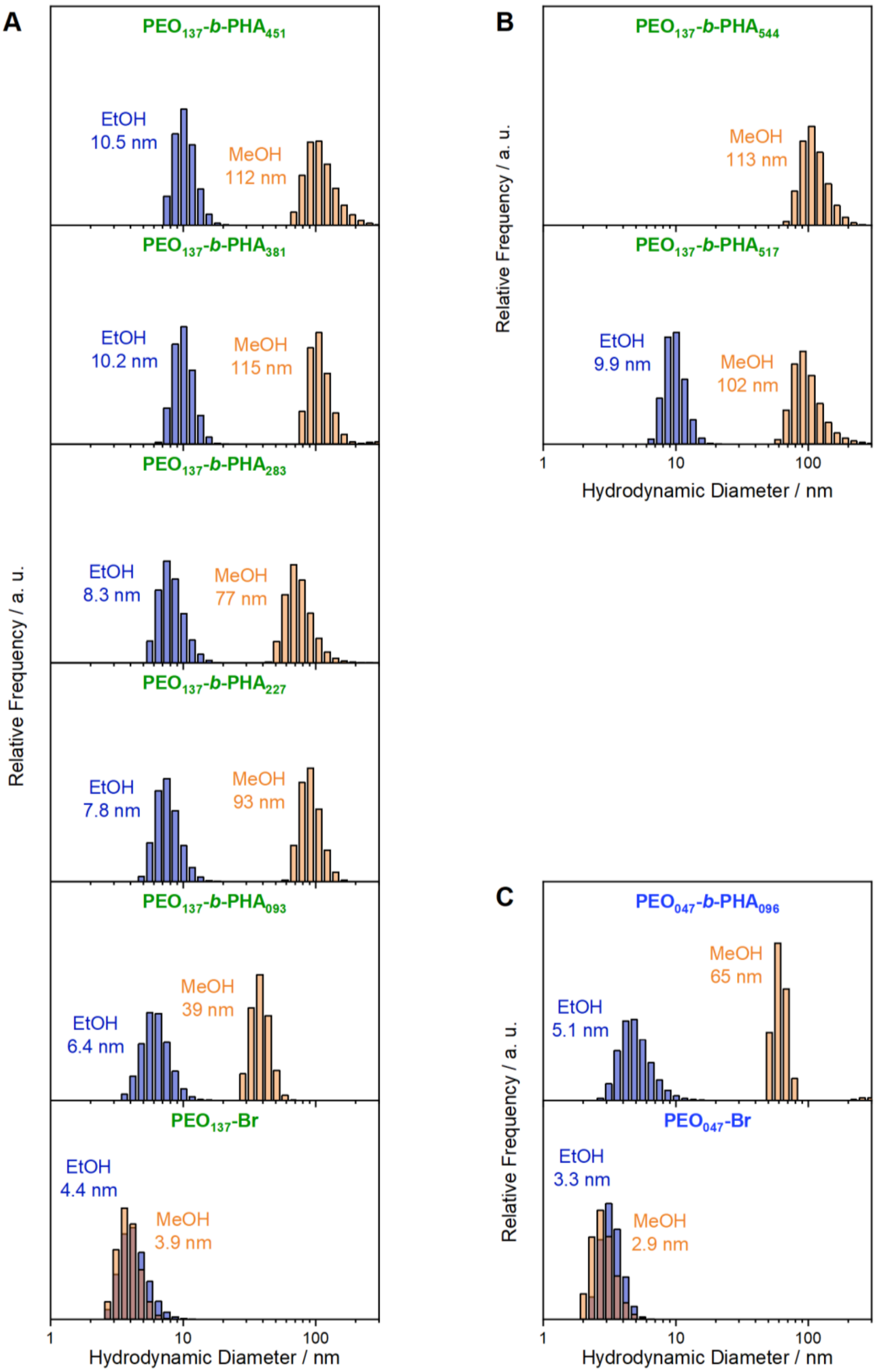

Figure S5. DLS data of PEO-Br macroinitiators (5 kDa and 2 kDa) and PEO-*b*-PHA block copolymers based on them in ethanol (blue) and in methanol (orange).

Table S4. Overview on all polymers prepared throughout this work including number-average molecular weight (M_n) from NMR, apparent number-average molecular weight (M_n^{app}) and polydispersity index ($Đ$) from GPC, and hydrodynamic size in ethanol and methanol from DLS.

Sample	M_n / kDa	M_n^{app} / kDa	$Đ$	d_{DLS}(EtOH) / nm	d_{DLS}(MeOH) / nm
**PEO_{441}-*b*-PHA_{390}**	80.5	84.6	1.51	11.6	101
**PEO_{441}-*b*-PHA_{326}**	70.5	74.0	1.50	10.4	96
**PEO_{441}-*b*-PHA_{270}**	61.7	68.0	1.52	11.7	86
**PEO_{441}-*b*-PHA_{173}**	46.6	57.3	1.13	10.2	62
**PEO_{372}-Br**	16.6	25.7	1.03	6.8	6.6
**PEO_{251}-*b*-PHA_{441}**	80.1	54.2	1.67	11.3	115
**PEO_{251}-*b*-PHA_{381}**	70.7	55.1	1.55	9.8	81
**PEO_{251}-*b*-PHA_{215}**	44.8	37.9	1.39	9.4	58
**PEO_{251}-*b*-PHA_{147}**	34.2	32.2	1.31	7.3	51
**PEO_{251}-*b*-PHA_{093}**	25.8	21.5	1.54	8.0	39
**PEO_{251}-Br**	11.3	15.7	1.03	5.7	5.3
**PEO_{137}-*b*-PHA_{544}**	91.1	53.9	1.60	–	113
**PEO_{137}-*b*-PHA_{517}**	86.9	50.3	1.57	9.9	102
**PEO_{137}-*b*-PHA_{451}**	76.6	50.0	1.57	10.5	112
**PEO_{137}-*b*-PHA_{381}**	65.7	47.9	1.45	10.2	115
**PEO_{137}-*b*-PHA_{283}**	50.4	39.5	1.44	8.3	77
**PEO_{137}-*b*-PHA_{227}**	41.7	29.8	1.54	7.8	93
**PEO_{137}-*b*-PHA_{093}**	20.7	19.2	1.41	6.4	39
**PEO_{137}-Br**	6.2	7.8	1.10	4.4	3.9
**PEO_{047}-*b*-PHA_{468}**	75.3	52.7	1.58	–	–
**PEO_{047}-*b*-PHA_{096}**	17.3	17.2	1.79	5.1	65
**PEO_{047}-Br**	2.3	3.2	1.04	3.3	2.9

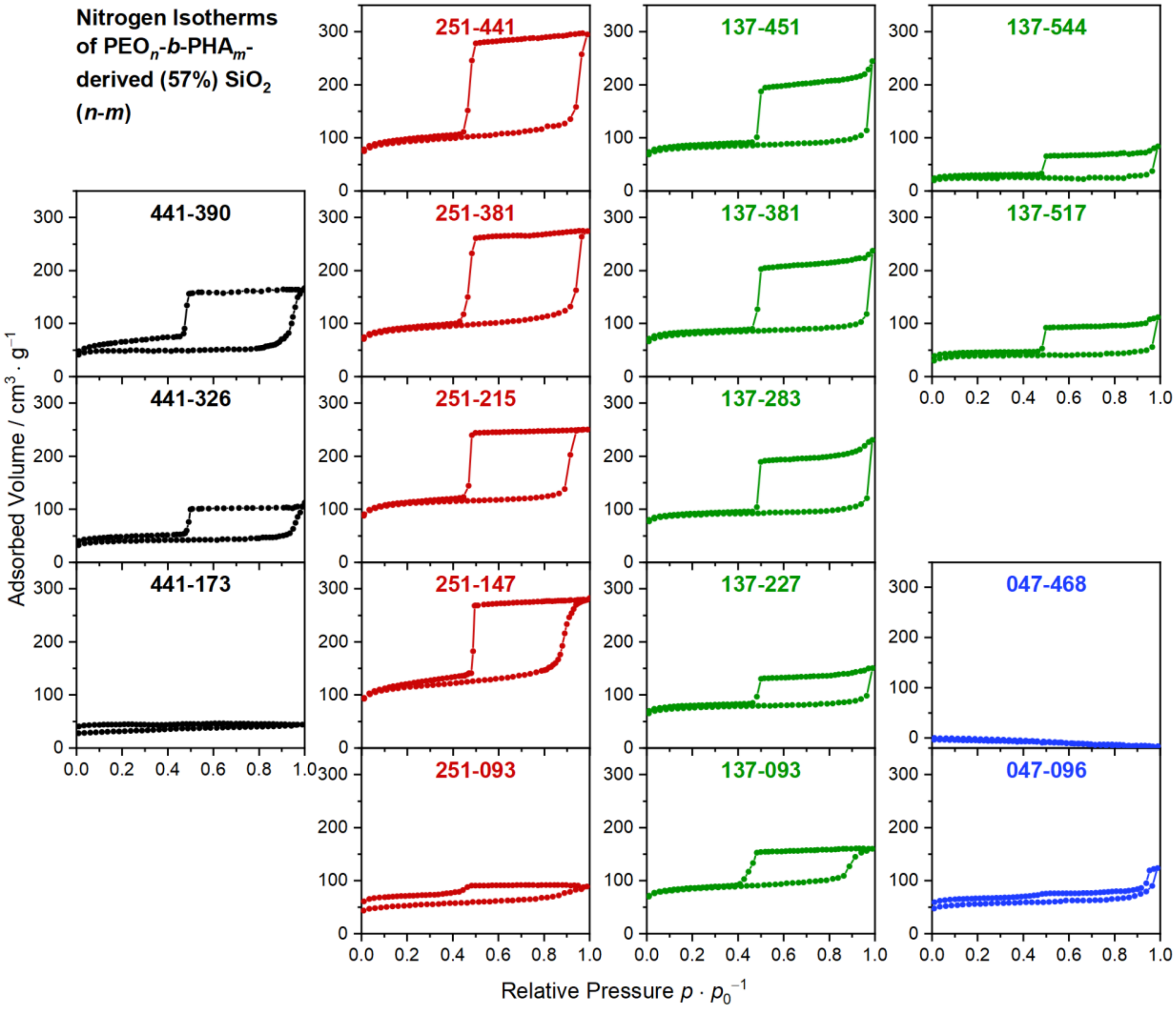

Figure S6. Nitrogen isotherms at 77 K of mesoporous silica prepared with 57 vol.% PEO$_n$-*b*-PHA$_m$ with a PEO block length of n = 441 (black), 251 (red), 137 (green), and 47 (blue) while the PHA block length m increases from the bottom to the top each.

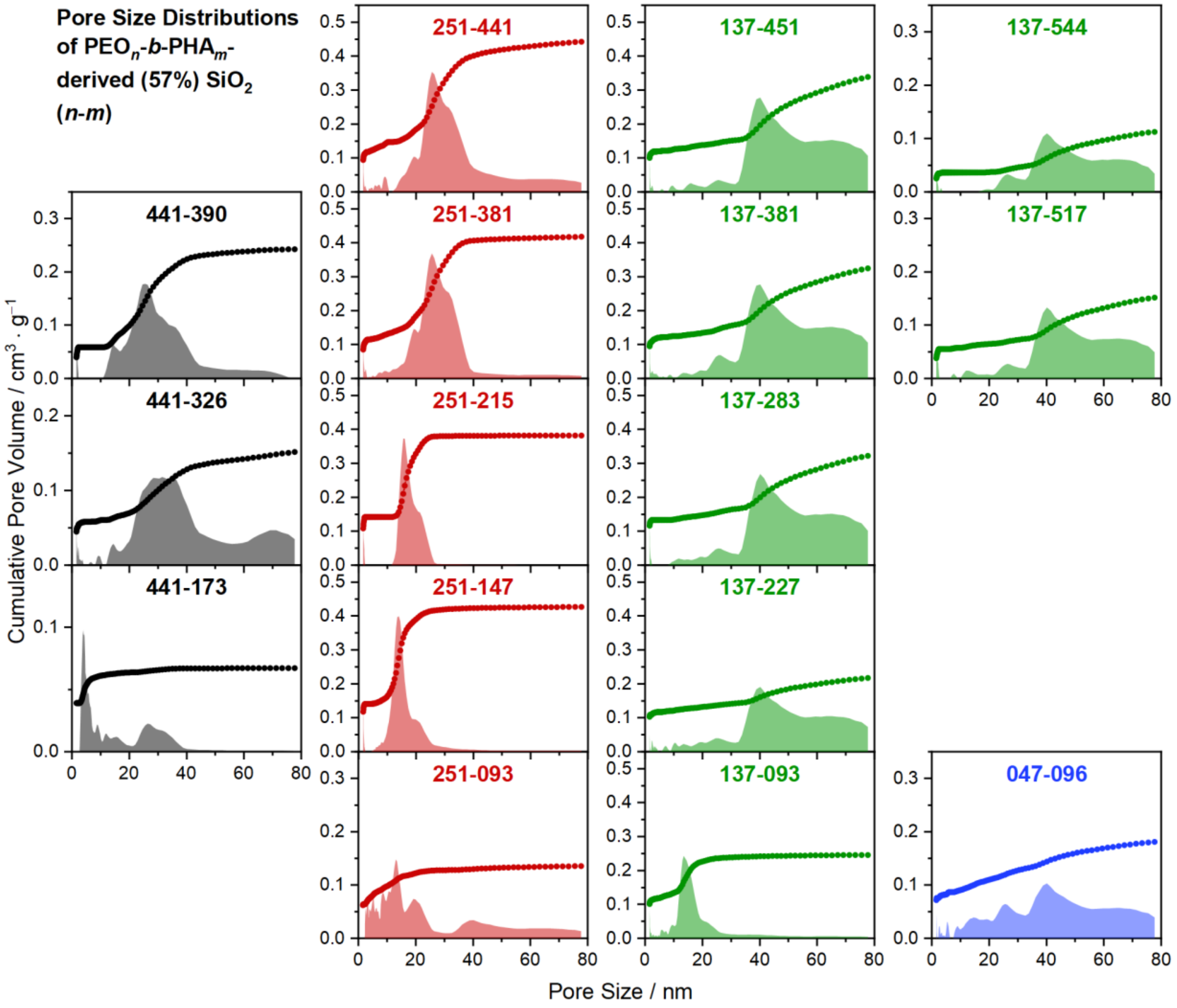

Figure S7. Pore size distributions of mesoporous silica prepared with 57 vol.% PEO$_n$-*b*-PHA$_m$ with a PEO block length of n = 441 (black), 251 (red), 137 (green), and 47 (blue) derived from the adsorption branch with an NLDFT kernel for cylindrical pores. The cumulative plot (filled symbols) is given in absolute values while the differential plot (filled area) is displayed in arbitrary units for eye guidance.

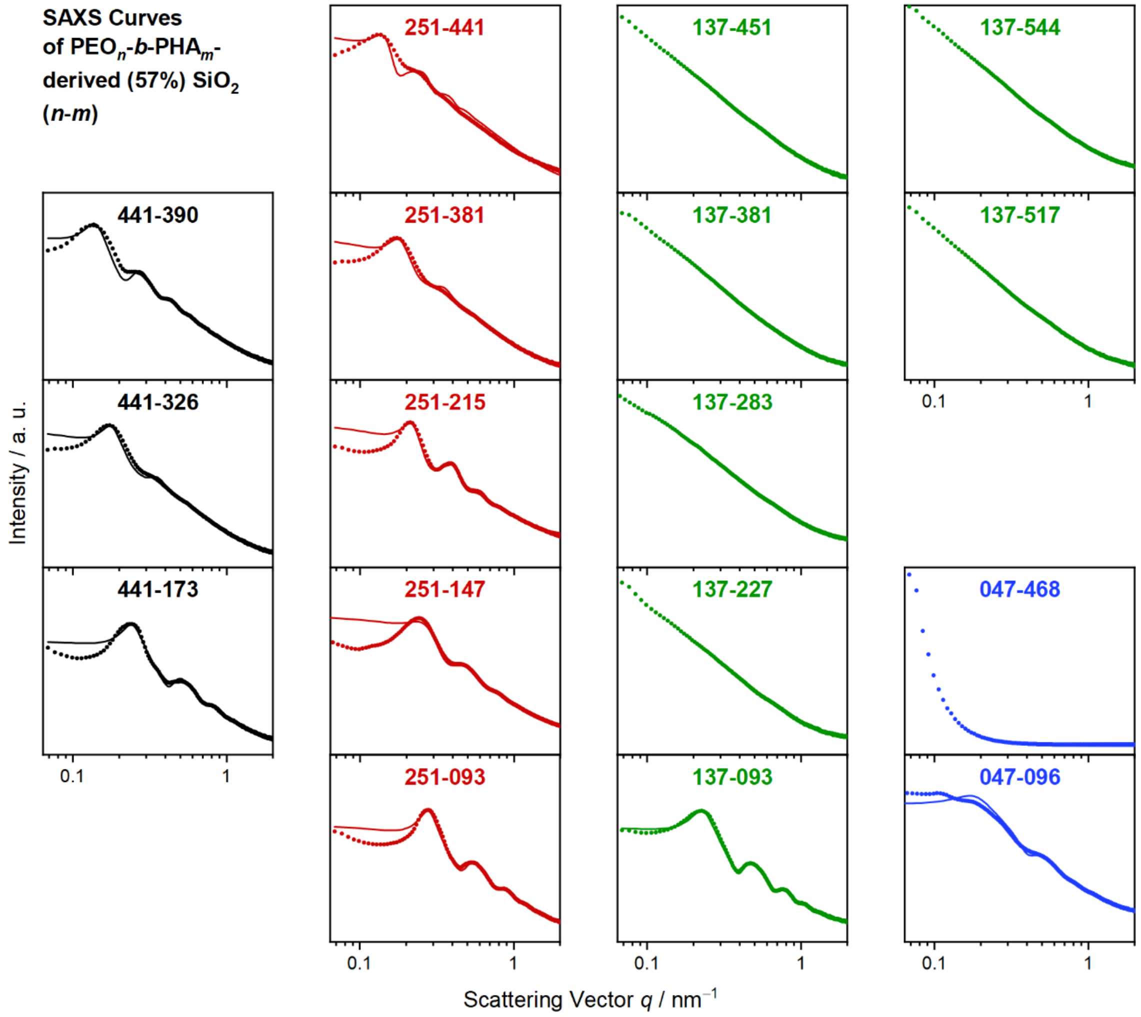

Figure S8. SAXS curves of mesoporous silica prepared with 57 vol.% PEO$_n$-*b*-PHA$_m$ with a PEO block length of n = 441 (black), 251 (red), 137 (green), and 47 (blue) while the PHA block length m increases from the bottom to the top each. Experimental data (dots) are fitted (solid lines) according to a Percus-Yevick model for spherical pores.

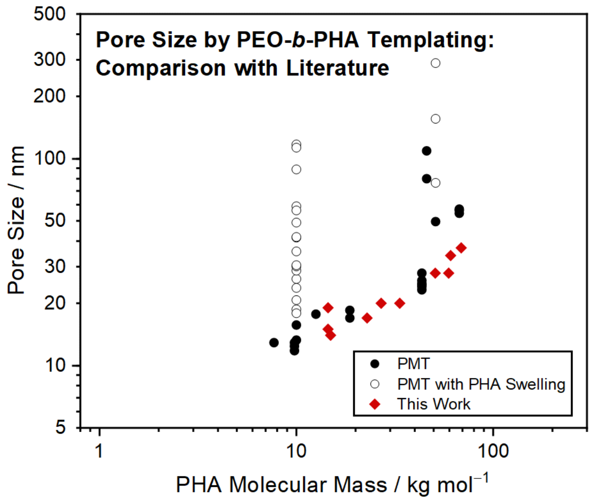

Figure S9. Comparison of the pore size of mesoporous materials obtained with PEO-*b*-PHA block copolymers of different PHA block lengths by soft templating with 57 vol.% template in this work (red diamonds) and by persistent micelle templating (PMT) without (filled black circles) and with PHA homopolymer swelling (open black circles) reported by Stefik and co-workers.[1–9] Note that some macroporous samples prepared by homopolymer swelling possess multimodal pore size distributions as summarized and categorized elsewhere.[1]

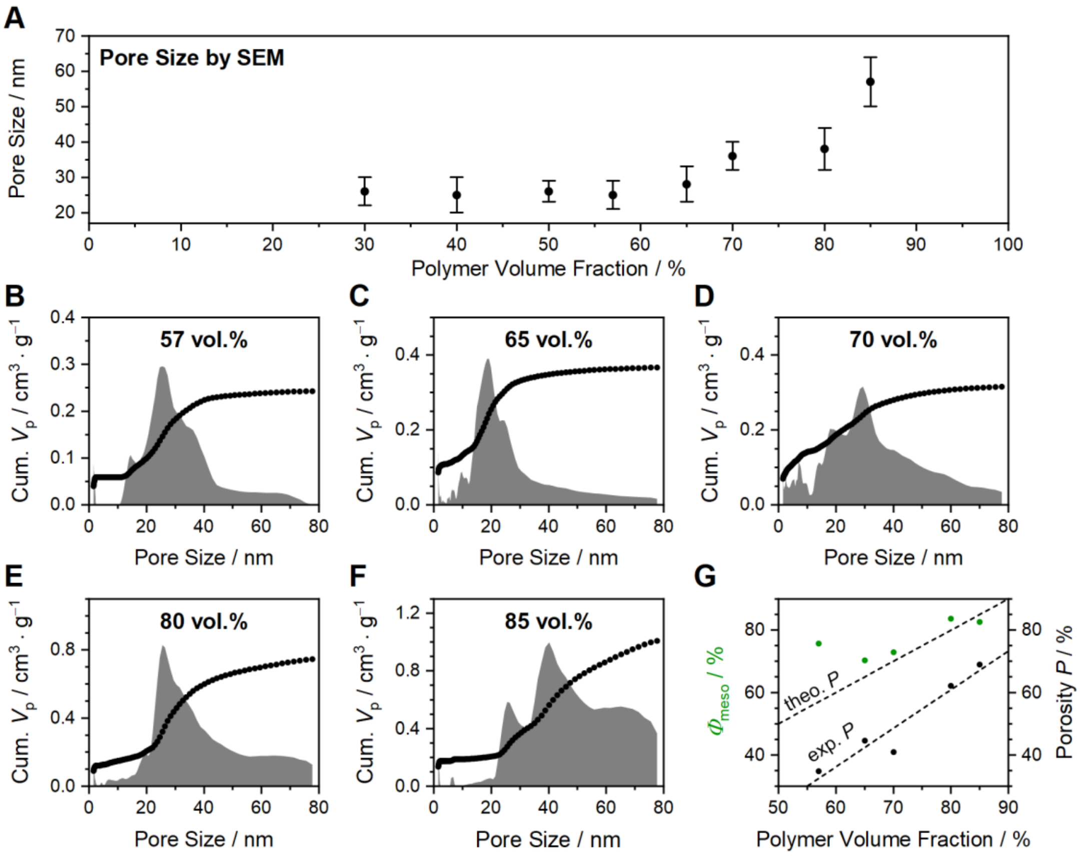

Figure S10. (A) Pore size and its standard deviation obtained from SEM of mesoporous silica prepared with different amounts of PEO_{441}-*b*-PHA_{270} as well as the pore size distribution derived from the adsorption branch of the nitrogen (77 K) isotherm with an NLDFT kernel for cylindrical pores for the samples prepared with (B) 57, (C) 65, (D) 70, (E) 80, (F) 85 vol.% soft template. (G) Experimental porosity (black dots) and share of the mesopore volume (green dots) compared to the total pore volume both obtained from physisorption in dependency on the polymer volume fraction. The experimental porosity follows a linear fit (lower dashed line) and is contrasted to the theoretical evolution (upper dashed line).

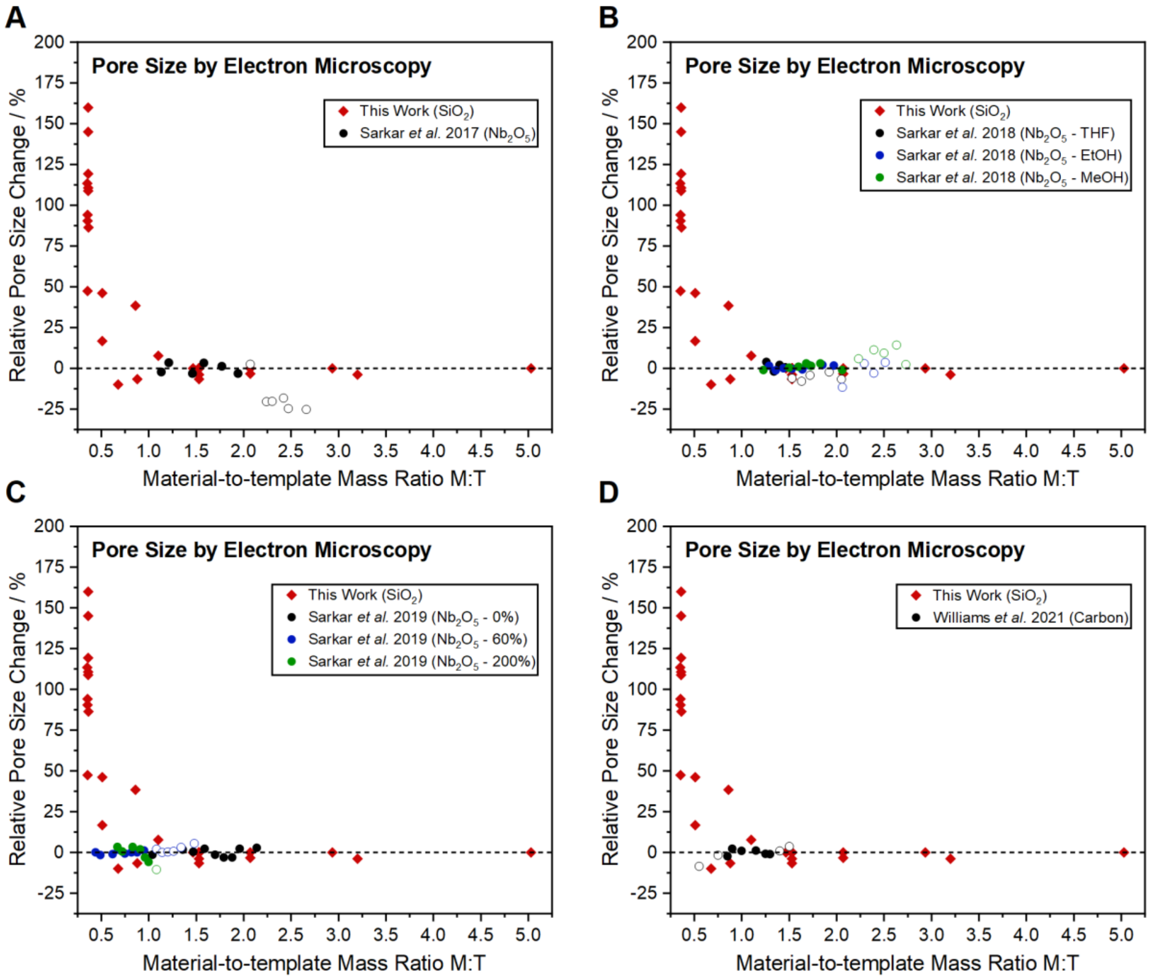

Figure S11. Comparison of relative pore size changes upon varying the template amount (the mass of final mesoporous material assuming full conversion in relation to the mass of soft template) observed in this study (red diamonds) to pore sizes found in literature for mesoporous materials templated with persistent micelles (filled circles) and dynamic micelles (open circles). The average pore diameter found by (A – C) SEM and (D) TEM in mesoporous niobia soft-templated with PEO-*b*-PHA (A) in tetrahydrofuran (THF),[4] (B) in THF, ethanol, and methanol, respectively,[6] and (C) with PEO-*b*-PHA swollen with different amounts of PHA homopolymer in ethanol[8] and (D) in mesoporous carbon templated with PEO-*b*-PS[10] are referenced to their respective pore size by persistent micelle templating and contrasted to SEM-based pore sizes (referenced to each pore size obtained with the lowest template amount) of mesoporous silica prepared in this study.

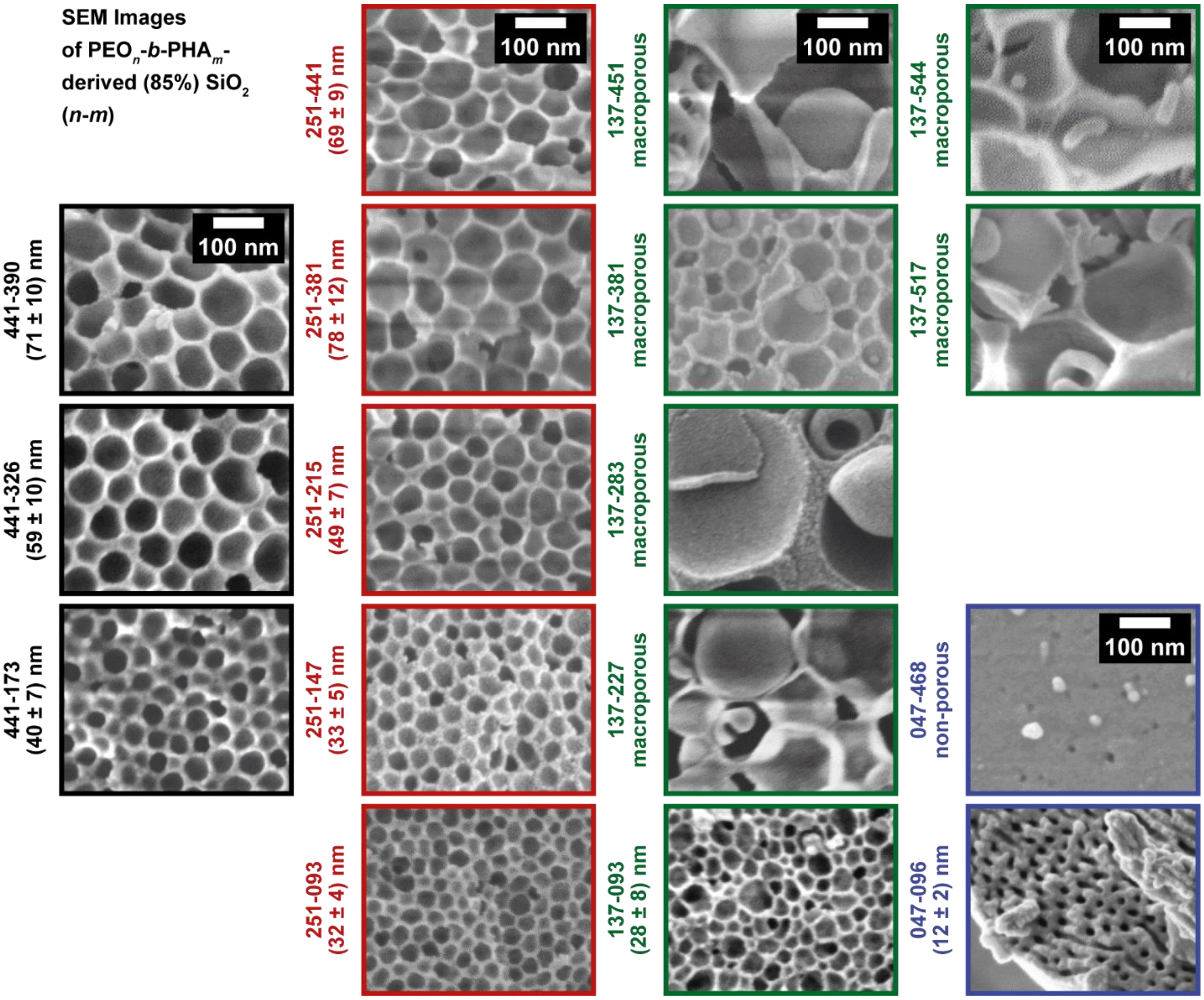

Figure S12. SEM images of mesoporous silica prepared with 85 vol.% PEO$_n$-b-PHA$_m$ with a PEO block length of n = 441 (black), 251 (red), 137 (green), and 47 (blue) while the PHA block length m increases from the bottom to the top each.

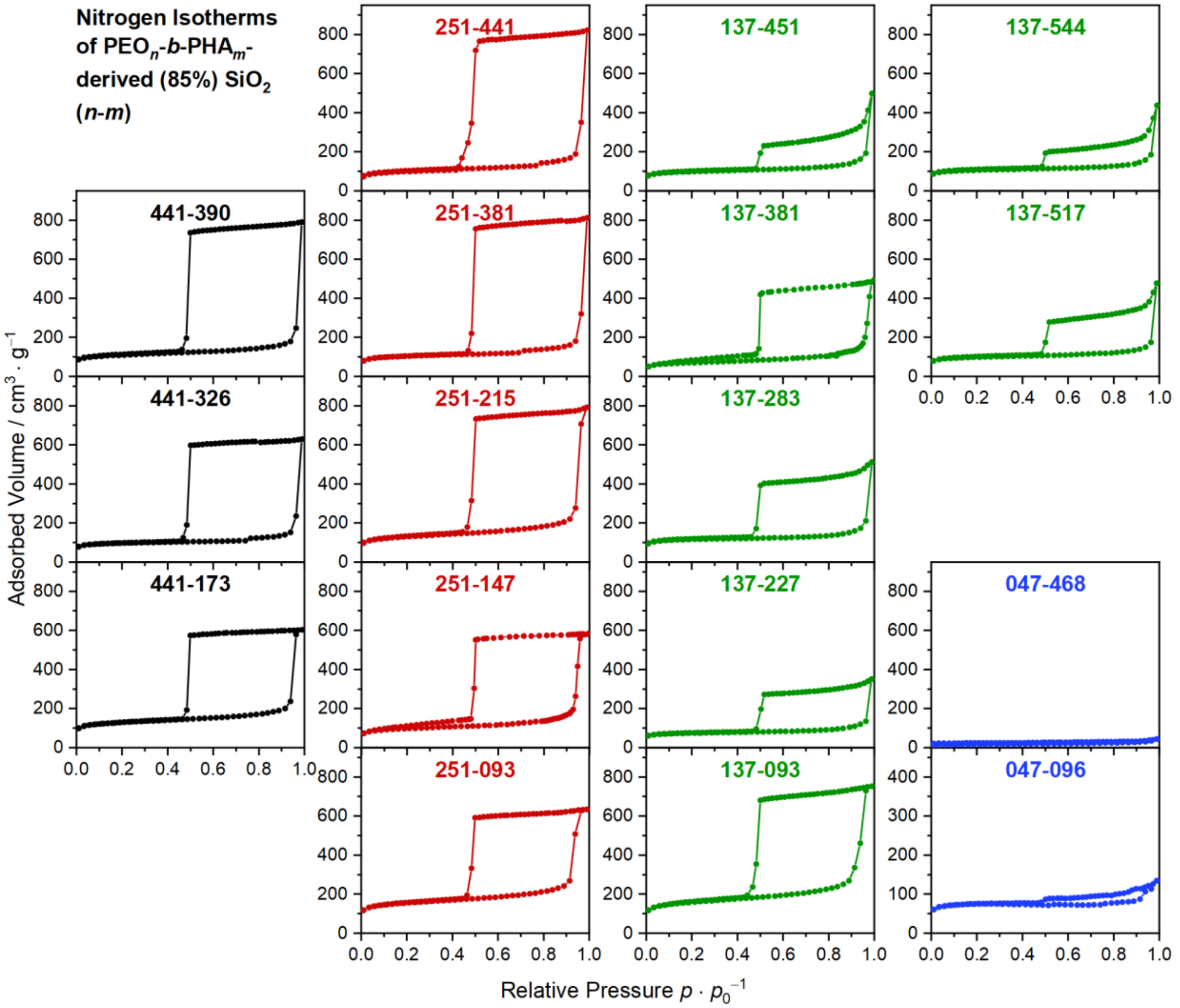

Figure S13. Nitrogen isotherms at 77 K of mesoporous silica prepared with 85 vol.% PEO$_n$-*b*-PHA$_m$ with a PEO block length of n = 441 (black), 251 (red), 137 (green), and 47 (blue) while the PHA block length m increases from the bottom to the top each.

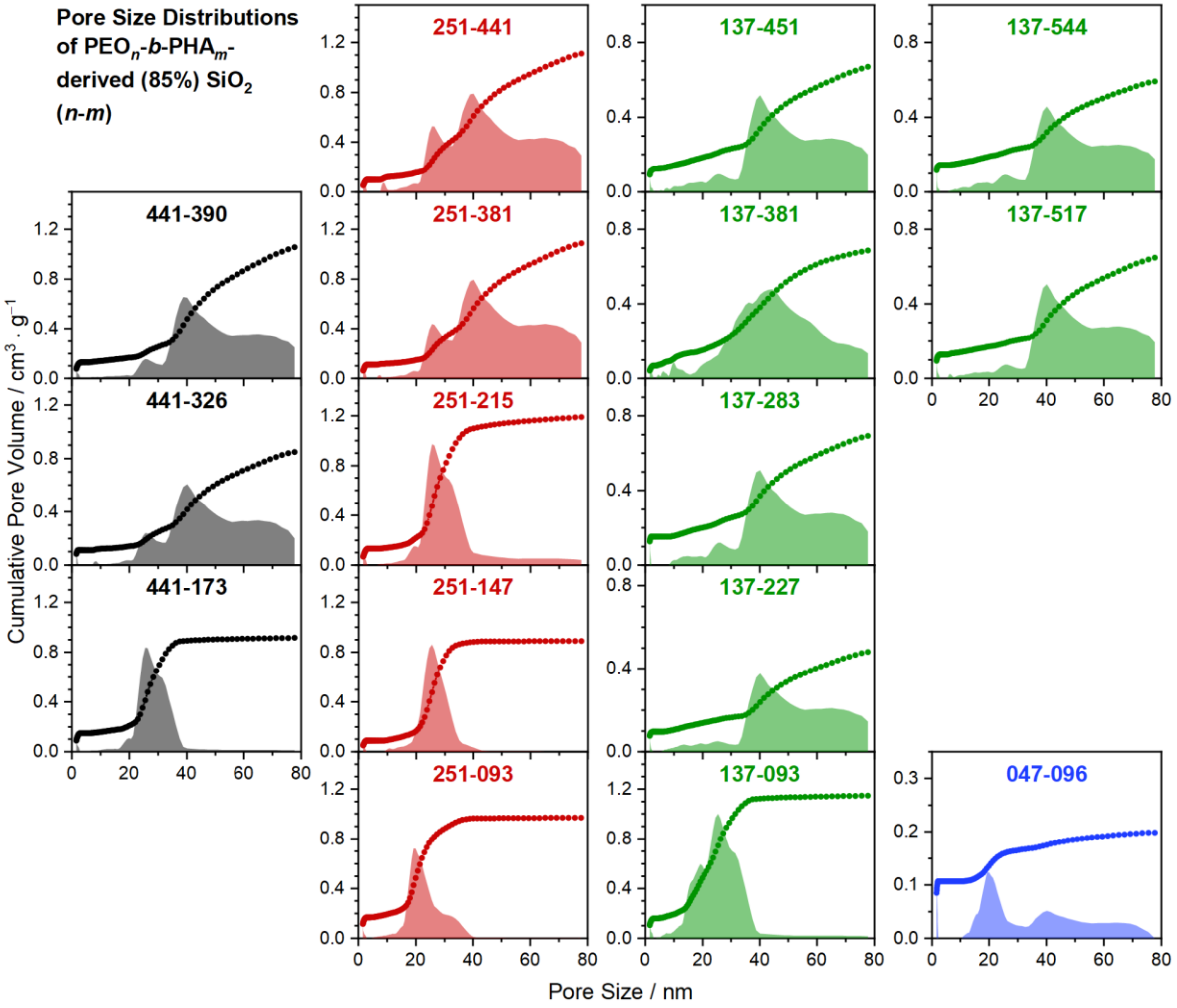

Figure S14. Pore size distributions of mesoporous silica prepared with 85 vol.% PEO$_n$-*b*-PHA$_m$ with a PEO block length of n = 441 (black), 251 (red), 137 (green), and 47 (blue) derived from the adsorption branch with an NLDFT kernel for cylindrical pores. The cumulative plot (filled symbols) is given in absolute values while the differential plot (filled area) is displayed in arbitrary units for eye guidance.

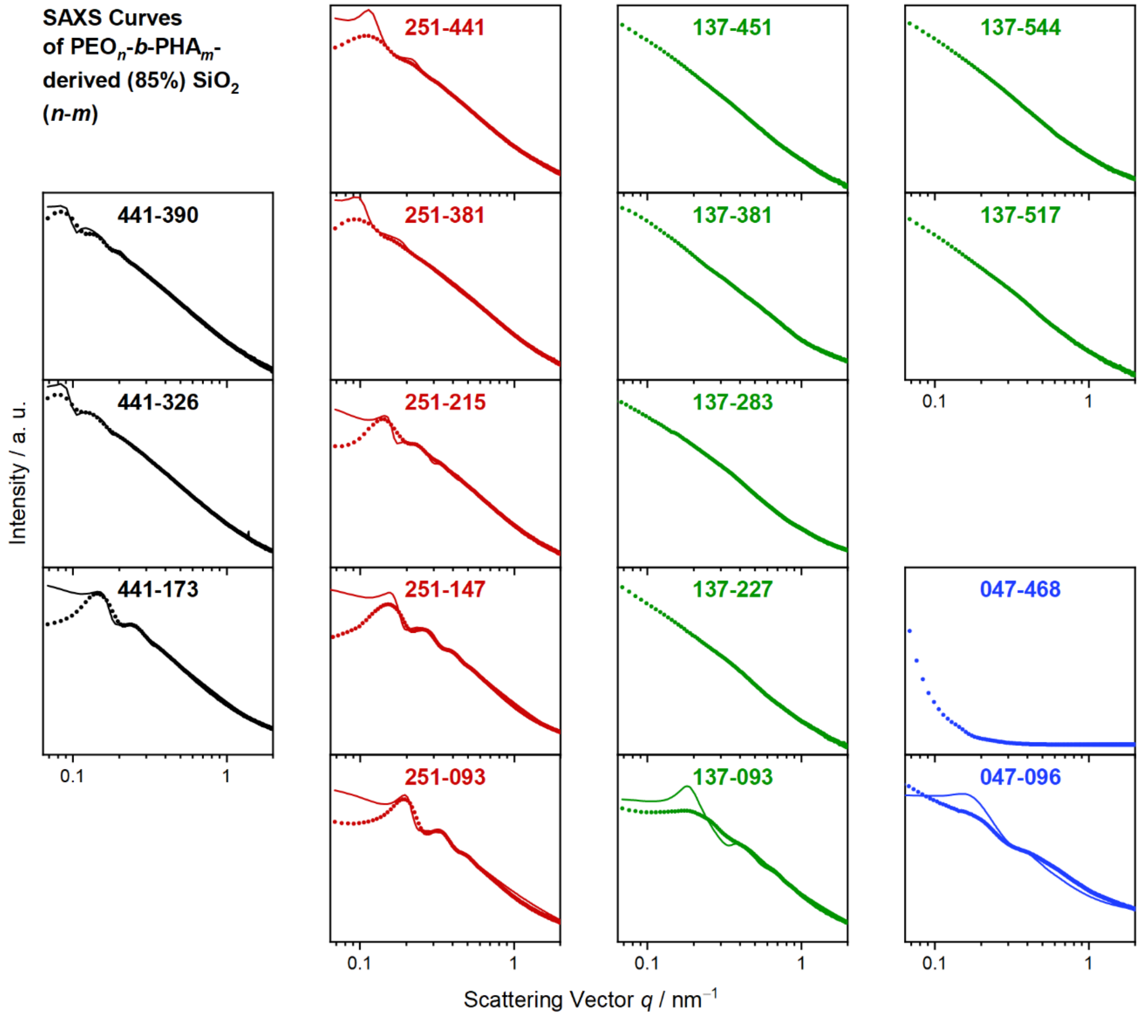

Figure S15. SAXS curves of mesoporous silica prepared with 85 vol.% PEO$_n$-*b*-PHA$_m$ with a PEO block length of n = 441 (black), 251 (red), 137 (green), and 47 (blue) while the PHA block length m increases from the bottom to the top each. Experimental data (dots) are fitted (solid lines) according to a Percus-Yevick model for spherical pores.

Table S5. Pore size (in nm) of all mesoporous silica powders possessing ordered arrays of spherical mesopores prepared with 57 and 85 vol.% soft template. Values from SEM were obtained by measuring the pore diameter of 50 pores and determining its standard deviation, values from nitrogen physisorption result from an NLDFT-based pore size distribution relying on the adsorption branch (mode value and full width at half maximum) after multiplication with a geometrical correction factor of 1.35 (spherical *versus* cylindrical pore geometry), and values from SAXS were received after fitting with a Percus-Yevick model for spherical pores.

	Pore Size (57 vol.%) / nm			Pore Size (85 vol.%) / nm		
Template	SEM	Phys	SAXS	SEM	Phys	SAXS
**PEO_{441}-*b*-PHA_{390}**	34 ± 6	34 ± 7	41 ± 10	71 ± 10	54 ± 8	85 ± 20
**PEO_{441}-*b*-PHA_{326}**	28 ± 3	43 ± 13	30 ± 10	59 ± 10	54 ± 8	85 ± 22
**PEO_{441}-*b*-PHA_{173}**	21 ± 2	–	21 ± 5	40 ± 7	38 ± 7	46 ± 14
**PEO_{251}-*b*-PHA_{441}**	37 ± 6	34 ± 4	49 ± 10	69 ± 9	54 ± 9	50 ± 22
**PEO_{251}-*b*-PHA_{381}**	28 ± 4	34 ± 6	31 ± 13	78 ± 12	54 ± 8	66 ± 26
**PEO_{251}-*b*-PHA_{215}**	20 ± 4	21 ± 3	30 ± 8	49 ± 7	39 ± 7	51 ± 13
**PEO_{251}-*b*-PHA_{147}**	17 ± 3	19 ± 3	23 ± 8	33 ± 5	34 ± 5	45 ± 11
**PEO_{251}-*b*-PHA_{093}**	15 ± 2	18 ± 3	20 ± 5	32 ± 4	26 ± 5	36 ± 10
**PEO_{137}-*b*-PHA_{093}**	19 ± 2	18 ± 3	23 ± 5	28 ± 8	29 ± 8	27 ± 9
**PEO_{047}-*b*-PHA_{096}**	14 ± 2	–	21 ± 8	12 ± 2	27 ± 4	26 ± 12

Table S6. Overview on the specific surface area S_{BET} of all silica samples (in $m^2\ g^{-1}$) as determined by the BET approach from the respective physisorption isotherm.

Soft Template	57 vol.%	85 vol.%	Soft Template	Φ	S_{BET} / $m^2\ g^{-1}$
**PEO$_{441}$-*b*-PHA$_{390}$**	200	410	**PEO$_{441}$-*b*-PHA$_{270}$**	30 vol.%	–
**PEO$_{441}$-*b*-PHA$_{326}$**	150	370		40 vol.%	–
**PEO$_{441}$-*b*-PHA$_{270}$**	240	540		50 vol.%	–
**PEO$_{441}$-*b*-PHA$_{173}$**	120	490		65 vol.%	330
**PEO$_{251}$-*b*-PHA$_{441}$**	350	370		70 vol.%	290
**PEO$_{251}$-*b*-PHA$_{381}$**	340	390		80 vol.%	400
**PEO$_{251}$-*b*-PHA$_{215}$**	430	480			
**PEO$_{251}$-*b*-PHA$_{147}$**	440	360			
**PEO$_{251}$-*b*-PHA$_{093}$**	200	580			
**PEO$_{137}$-*b*-PHA$_{544}$**	100	410			
**PEO$_{137}$-*b*-PHA$_{517}$**	150	380			
**PEO$_{137}$-*b*-PHA$_{451}$**	320	370			
**PEO$_{137}$-*b*-PHA$_{381}$**	310	260			
**PEO$_{137}$-*b*-PHA$_{283}$**	350	450			
**PEO$_{137}$-*b*-PHA$_{227}$**	300	280			
**PEO$_{137}$-*b*-PHA$_{093}$**	330	580			
**PEO$_{047}$-*b*-PHA$_{468}$**	–	60			
**PEO$_{047}$-*b*-PHA$_{096}$**	220	290			

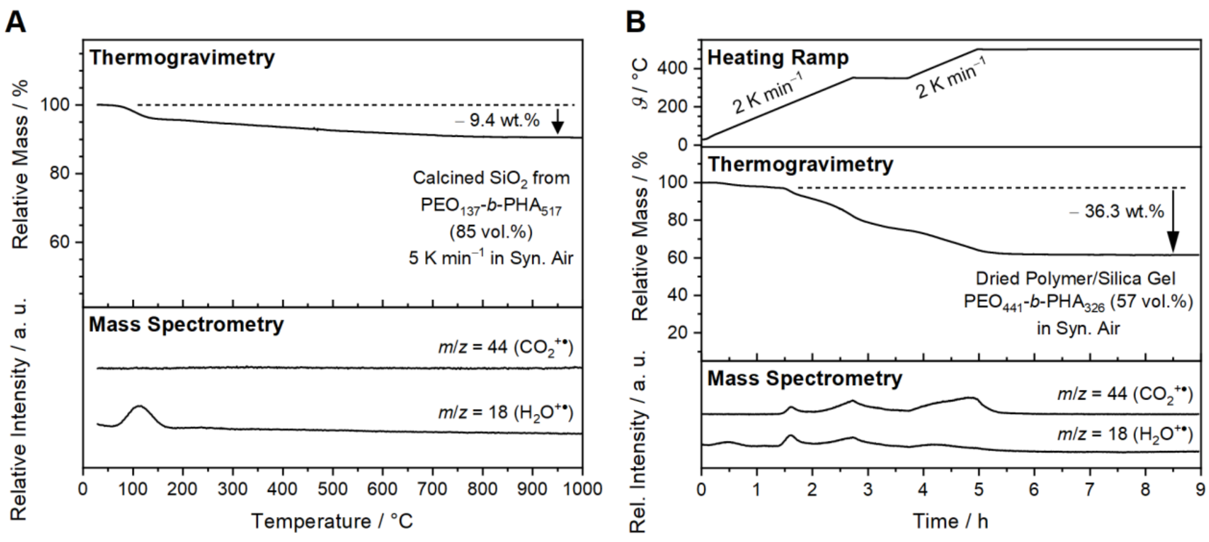

Figure S16. Thermogravimetric curve of (A) mesoporous silica prepared with 85 vol.% PEO_{137}-*b*-PHA_{517} measured in synthetic air with a heating ramp of 5 K min^{-1} and (B) of a dried PEO-*b*-PHA/silica hybrid gel with 57 vol.% PEO_{441}-*b*-PHA_{326} measured in synthetic air according to the heating ramp used for all syntheses. In addition, the intensity evolution of the mass signals corresponding to an *m*/*z* ratio of 18 (water) and 44 (CO_2) are shown below (note that all data in (B) are plotted against the elapsed time leading to discontinuances at each dwell time of the heating ramp).

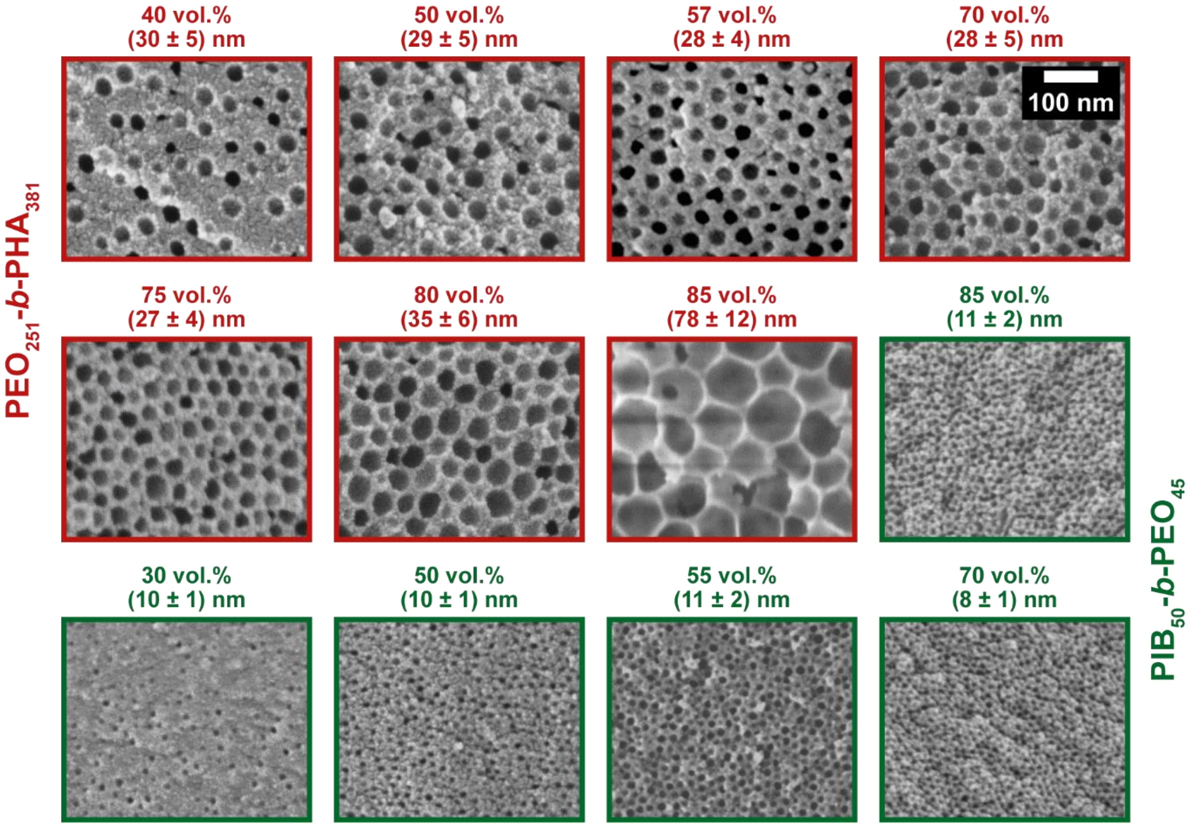

Figure S17. SEM images of mesoporous silica prepared with different amounts of PEO_{251}-*b*-PHA_{381} (red) and PIB_{50}-*b*-PEO_{45} (green), respectively. The respective polymer amount and resulting pore size is displayed above each image.

Since the volume of a material can be determined by dividing its mass by its density, the template concentration sometimes given in literature as the mass ratio (M:T) of the oxide assuming full conversion of the precursor to the mass of the soft template used can be converted to the polymer volume fraction Φ by equation **(S1)** if the density of the oxide ρ_{oxide} and soft template ρ_{BCP} are known:

$$\Phi = \frac{1}{1 + (\text{M:T})\,\frac{\rho_{\text{BCP}}}{\rho_{\text{oxide}}}} \qquad \textbf{(S1)}$$

REFERENCES

(1) Stefik, M. Single-Variable Porous Nanomaterial Series from Polymer Structure-Directing Agents. *J. Mater. Res.* **2022**, *37*, 25–42.

(2) Lokupitiya, H. N.; Jones, A.; Reid, B.; Guldin, S.; Stefik, M. Ordered Mesoporous to Macroporous Oxides with Tunable Isomorphic Architectures: Solution Criteria for Persistent Micelle Templates. *Chem. Mater.* **2016**, *28*, 1653–1667.

(3) Peters, K.; Lokupitiya, H. N.; Sarauli, D.; Labs, M.; Pribil, M.; Rathouský, J.; Kuhn, A.; Leister, D.; Stefik, M.; Fattakhova-Rohlfing, D. Nanostructured Antimony-Doped Tin Oxide Layers with Tunable Pore Architectures as Versatile Transparent Current Collectors for Biophotovoltaics. *Adv. Funct. Mater.* **2016**, *26*, 6682–6692.

(4) Sarkar, A.; Stefik, M. How to Make Persistent Micelle Templates in 24 Hours and Know It Using X-Ray Scattering. *J. Mater. Chem. A* **2017**, *5*, 11840–11853.

(5) Lokupitiya, H. N.; Stefik, M. Cavitation-Enabled Rapid and Tunable Evolution of High-χN Micelles as Templates for Ordered Mesoporous Oxides. *Nanoscale* **2017**, *9*, 1393–1397.

(6) Sarkar, A.; Evans, L.; Stefik, M. Expanded Kinetic Control for Persistent Micelle Templates with Solvent Selection. *Langmuir* **2018**, *34*, 5738–5749.

(7) Lantz, K. A.; Clamp, N. B.; van den Bergh, W.; Sarkar, A.; Stefik, M. Full Gamut Wall Tunability from Persistent Micelle Templates via Ex Situ Hydrolysis. *Small* **2019**, *15*, 1–10.

(8) Sarkar, A.; Thyagarajan, A.; Cole, A.; Stefik, M. Widely Tunable Persistent Micelle Templates via Homopolymer Swelling. *Soft Matter* **2019**, *15*, 5193–5203.

(9) Bergh, W.; Lokupitiya, H. N.; Vest, N. A.; Reid, B.; Guldin, S.; Stefik, M. Nanostructure Dependence of T-Nb_2O_5 Intercalation Pseudocapacitance Probed Using Tunable Isomorphic Architectures. *Adv. Funct. Mater.* **2021**, *31*, 2007826.

(10) Williams, E. R.; McMahon, P. L.; Reynolds, J. E.; Snider, J. L.; Stavila, V.; Allendorf, M. D.; Stefik, M. Tailored Porous Carbons Enabled by Persistent Micelles with Glassy Cores. *Mater. Adv.* **2021**, *2*, 5381–5395.
